# Coreceptor therapy has distinct short- and long-term tolerogenic effects intrinsic to autoreactive effector T cells

**DOI:** 10.1172/jci.insight.149130

**Published:** 2021-09-08

**Authors:** Matthew Clark, Charles J. Kroger, Qi Ke, Rui Zhang, Karen Statum, J. Justin Milner, Aaron Martin, Bo Wang, Roland Tisch

**Affiliations:** 1 Department of Microbiology and Immunology, and; 2 Lineberger Comprehensive Cancer Center, University of North Carolina at Chapel Hill School of Medicine, Chapel Hill, North Carolina, USA.

**Keywords:** Autoimmunity, Immunology, Diabetes, Immunotherapy, T cells

## Abstract

Immunotherapies are needed in the clinic that effectively suppress β cell autoimmunity and reestablish long-term self-tolerance in type 1 diabetes. We previously demonstrated that nondepleting anti-CD4 (αCD4) and αCD8α antibodies establish rapid and indefinite remission in recent-onset diabetic NOD mice. Diabetes reversal by coreceptor therapy (CoRT) is induced by suppression of pathogenic effector T cells (Teffs) and the selective egress of T cells from the pancreatic lymph nodes and islets that remain free of infiltration in the long term. Here, we defined CoRT-induced events regulating early Teff function and pancreatic residency, and long-term tolerance. TCR-driven gene expression controlling autoreactive Teff expansion and proinflammatory activity was suppressed by CoRT, and islet T cell egress was dependent on sphingosine-1 phosphate. In both murine and human T cells, CoRT upregulated the Foxo1 transcriptional axis, which in turn was required for suppression and efficient pancreatic egress of Teffs. Interestingly, long-term tolerance induced in late-preclinical NOD mice was marked by reseeding of the pancreas by a reduced CD8^+^ Teff pool exhibiting an exhausted phenotype. Notably, PD-1 blockade, which rescues exhausted Teffs, resulted in diabetes onset in protected animals. These findings demonstrate that CoRT has distinct intrinsic effects on Teffs that impact events early in induction and later in maintenance of self-tolerance.

## Graphical abstract

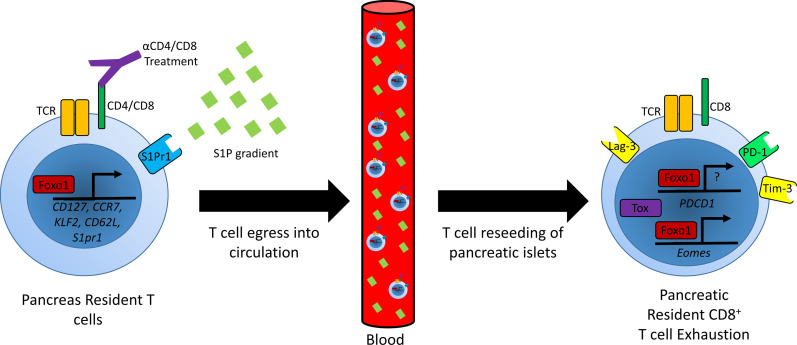

## Introduction

Type 1 diabetes (T1D) is an autoimmune disease leading to destruction and/or dysregulation of the insulin-producing β cells of the pancreatic islets of Langerhans (1–4). A role for T cells in driving human islet pathology is supported by a number of findings. For instance, the strongest genetic association with T1D susceptibility maps to specific HLA class I and II haplotypes (5, 6). Furthermore, the islets of T1D individuals are often infiltrated with CD4^+^, and particularly CD8^+^, T cells (7, 8). Moreover, a broader and more robust β cell–specific T cell response, typically marked by a type 1 IFN-γ^+^ phenotype (i.e., Th1, Tc1), is associated with the aggressive T1D that develops during childhood versus adult onset of diabetes (9, 10). In addition, an inverse correlation between the frequency of exhausted β cell–specific CD8^+^ T cells in blood and the pace of T1D progression has recently been reported (11). In NOD mice, a spontaneous model of T1D, CD4^+^ and CD8^+^ effector T cells (Teffs) are the primary mediators of β cell destruction (12). β Cell autoimmunity in NOD mice is marked by progressive infiltration of the islets (i.e., insulitis) by CD4^+^ and CD8^+^ T cells and other immune effectors (13–16). Once approximately 80% of β cell mass has been destroyed or rendered nonfunctional, overt diabetes is established (17).

There continues to be an urgent need for immunotherapies that reestablish long-term β cell self-tolerance for the prevention and treatment of T1D in the clinic. Arguably, the most effective clinical immunotherapy for T1D has been the administration of nonmitogenic anti-CD3 (αCD3) Ab (18, 19). Treatment of newly diagnosed T1D patients with αCD3 protects residual β cell mass up to 4 years in some individuals, although diabetes reversal is not achieved (20). Furthermore, the transient reduction in systemic T cells by αCD3 makes dosing and subsequent interventions problematic, and has been linked to recurrent viral infections (21, 22). Moreover, αCD3 binding to the T cell receptor (TCR) complex can activate T cells, resulting in cytokine release, inflammation, and activation-induced cell death (AICD) of T cells (23, 24). Nevertheless, these findings provide important evidence that progression of human T1D can be modulated by targeting T cells.

The use of nondepleting (ND) Ab provides a strategy to modulate the function of Teffs while avoiding systemic T cell depletion. Our group has previously reported that treatment of newly diabetic NOD mice with a short course of ND αCD4 and αCD8α (ND αCD4/αCD8α) reverses clinical T1D indefinitely without impacting protective adaptive immunity (25). Induction of T1D remission by coreceptor therapy (CoRT) is accompanied by the rapid suppression of Teff function and the selective egress of T cells (independent of apoptosis induction) from the islets and pancreatic draining lymph node (PLN) (25–27). The latter is in part due to ND αCD4/αCD8α–mediated suppression of IFN-γ secretion by Teffs, which blocks a feed-forward loop needed for expression of local retention cues such as CXCL9 and CXCL10 (27). The factors controlling tissue residency in chronic autoimmune settings, such as T1D, remain undefined. Similarly, the molecular events rapidly induced by ND αCD4/αCD8α binding that modulate Teff tissue residency and function are poorly understood.

The Foxo1 transcriptional axis plays a critical role in regulating various aspects of T cell immunobiology, including T cell homeostasis, effector/memory function, and trafficking (28). In resting T cells, Foxo1 is constitutively expressed and active, driving the transcription of multiple genes including *Klf2*, *Ccr7*, and *Cd127* (29). KLF2 promotes the transcription of *Sell* (CD62L) and *S1pr1*, which encodes the receptor for sphingosine-1-phosphate (S1P), a critical mediator of T cell tissue egress (30). Therefore, the Foxo1 transcriptional axis establishes a promigratory phenotype that enables quiescent T cells to circulate systemically in search of cognate antigen. Upon antigen recognition and TCR signaling, activated AKT phosphorylates Foxo1, which in turn leads to Foxo1 nuclear exclusion and degradation (31, 32). Notably, Foxo1 inhibits the function of the transcription factor T-bet, which is required for Teff differentiation and maintenance (33, 34). Furthermore, Foxo1 regulates events involved in formation of exhausted T cells, a process that affects T1D progression (35–37).

In this report, we demonstrate that in addition to inducing remission, CoRT effectively prevents diabetes onset when administered at a late preclinical T1D stage in NOD mice. Importantly, CoRT has short- and long-term tolerogenic effects that intrinsically impact the pathogenicity of β cell–specific Teffs. Foxo1 plays a key role in the early events governing CoRT-induced T cell tolerance. Dampened TCR signaling after ND αCD4/αCD8α binding to CD4^+^ and CD8^+^ Teffs results in enhanced Foxo1 transcriptional activity, upregulation of S1PR1, and subsequent S1P-dependent pancreatic egress of murine and human T cells in NOD and humanized mice, respectively. Importantly, pancreatic Teffs lacking Foxo1 expression continue to exhibit a type 1 phenotype coupled with reduced egress following CoRT. Upregulation of Foxo1 by ND αCD4/αCD8α is dependent on the magnitude of TCR signaling in vivo. Finally, CoRT given at a late preclinical T1D stage establishes long-term self-tolerance via selective induction of exhaustion within a reduced pool of CD8^+^ Teffs that reseed the pancreas over time. In summary, evidence is provided that defines what we believe is novel crosstalk between the CD4 and CD8 coreceptor molecules, TCR signaling, and the Foxo1 transcriptional axis, and that CoRT limits the intrinsic pathogenicity of autoreactive CD8^+^ Teffs in the long term. Importantly, these results also support the clinical application of ND αCD4/αCD8α for prevention and treatment of T1D and other T cell–driven pathologies.

## Results

### CoRT reverses and prevents diabetes onset at a late preclinical T1D stage.

**Figure 1:**
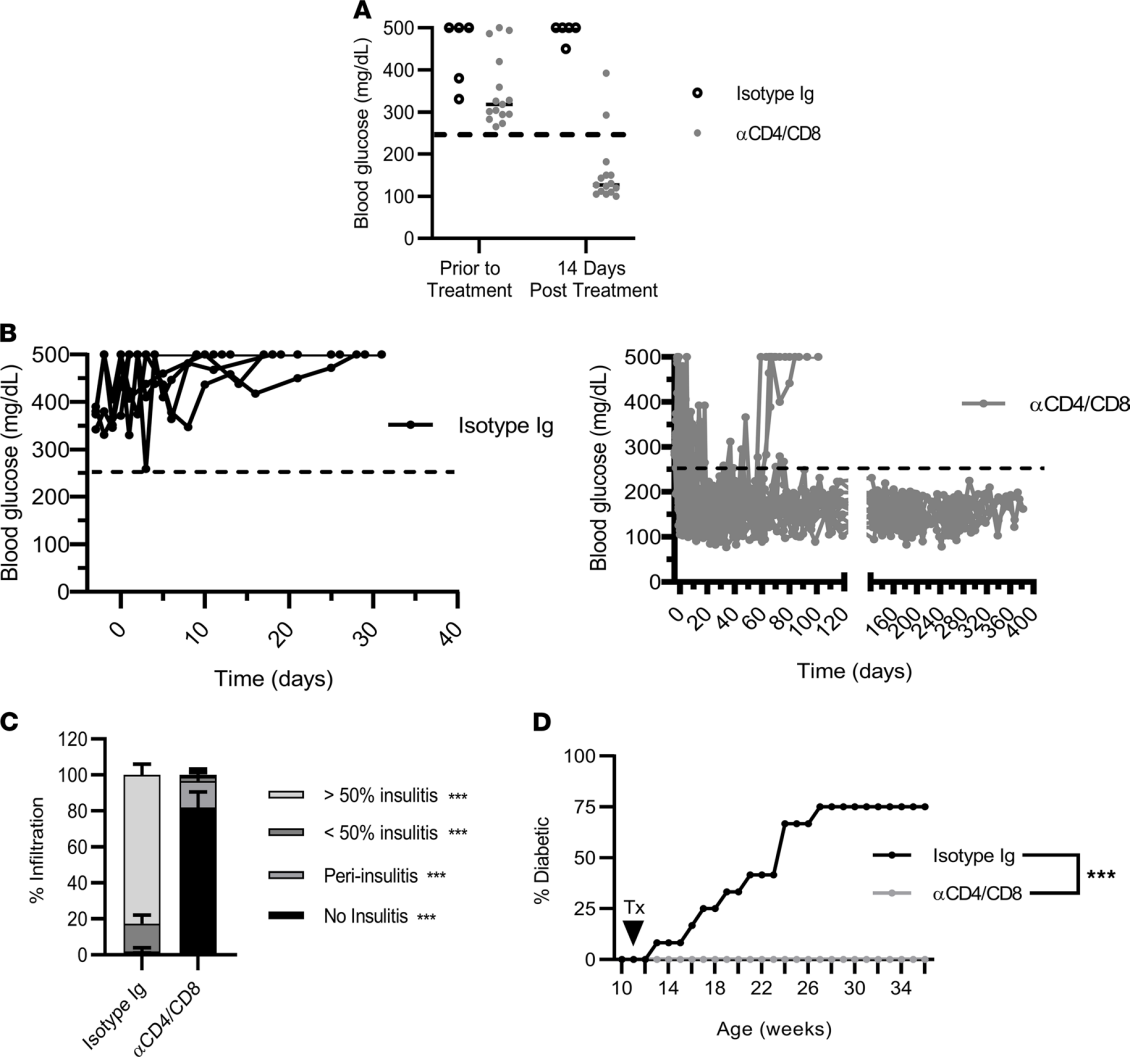
ND αCD4/αCD8α Abs reverse diabetes in new-onset NOD mice and prevent diabetes development in NOD nice treated at a late preclinical stage. (**A** and **B**) Blood glucose levels from diabetic NOD female mice (**A**) 14 days and (**B**) up to 390 days after treatment with 2 injections 1 day apart with ND αCD4/αCD8α (*n* = 15) or isotype control Ab (*n* = 5). (**C**) Insulitis from diabetic NOD female mice 14 days after control Ab (*n* = 5) and up to 390 days after ND αCD4/αCD8α (*n* = 5). (**D**) Twelve-week-old NOD female were treated with 2 injections 1 day apart with ND αCD4/αCD8α (*n* = 11) or control Ab (*n* = 12), and diabetes onset was monitored. ***P < 0.001, determined by Student’s *t* test (**C**) or Kaplan-Meier (**D**). Error bars depict SD. The data are representative of 2 or more experiments.

Consistent with our earlier findings, a short course of both ND αCD4 (clone YTS177) and ND αCD8α (clone YTS105) induced diabetes reversal in 87% (13/15) of NOD mice by 14 days after treatment (Figure 1A) (25, 27). Once established, remission was maintained up to 400 days after treatment, with the majority of islets remaining free of insulitis (Figure 1, B and C).

Various immunotherapies are effective at only a particular stage of disease progression in NOD mice (38). For instance, αCD3 therapy induces remission but has been reported to have only minimal efficacy at preventing diabetes when administered to preclinical NOD mice (39). To test whether CoRT reestablishes β cell tolerance at a late preclinical T1D stage, 12-week-old NOD female mice were treated with 2 injections of YTS177 and YTS105 over 2 days, and diabetes was monitored up to 36 weeks of age. At 12 weeks of age, β cell autoimmunity is well established in NOD female mice, and the islets are heavily infiltrated. As expected, the majority (9/12; 75%) of NOD mice receiving isotype control Ab (clone 2A3) developed diabetes (Figure 1D). In contrast, none (0/11) of the ND αCD4/αCD8α–treated NOD mice became overtly diabetic (Figure 1D). Together, these findings demonstrate that CoRT is effective at suppressing ongoing β cell autoimmunity and reestablishing long-term tolerance at both late-preclinical and clinical stages of T1D in NOD mice.

### CoRT transcriptionally reprograms diabetogenic T cells in vivo to decrease effector function and tissue residency.

**Figure 2:**
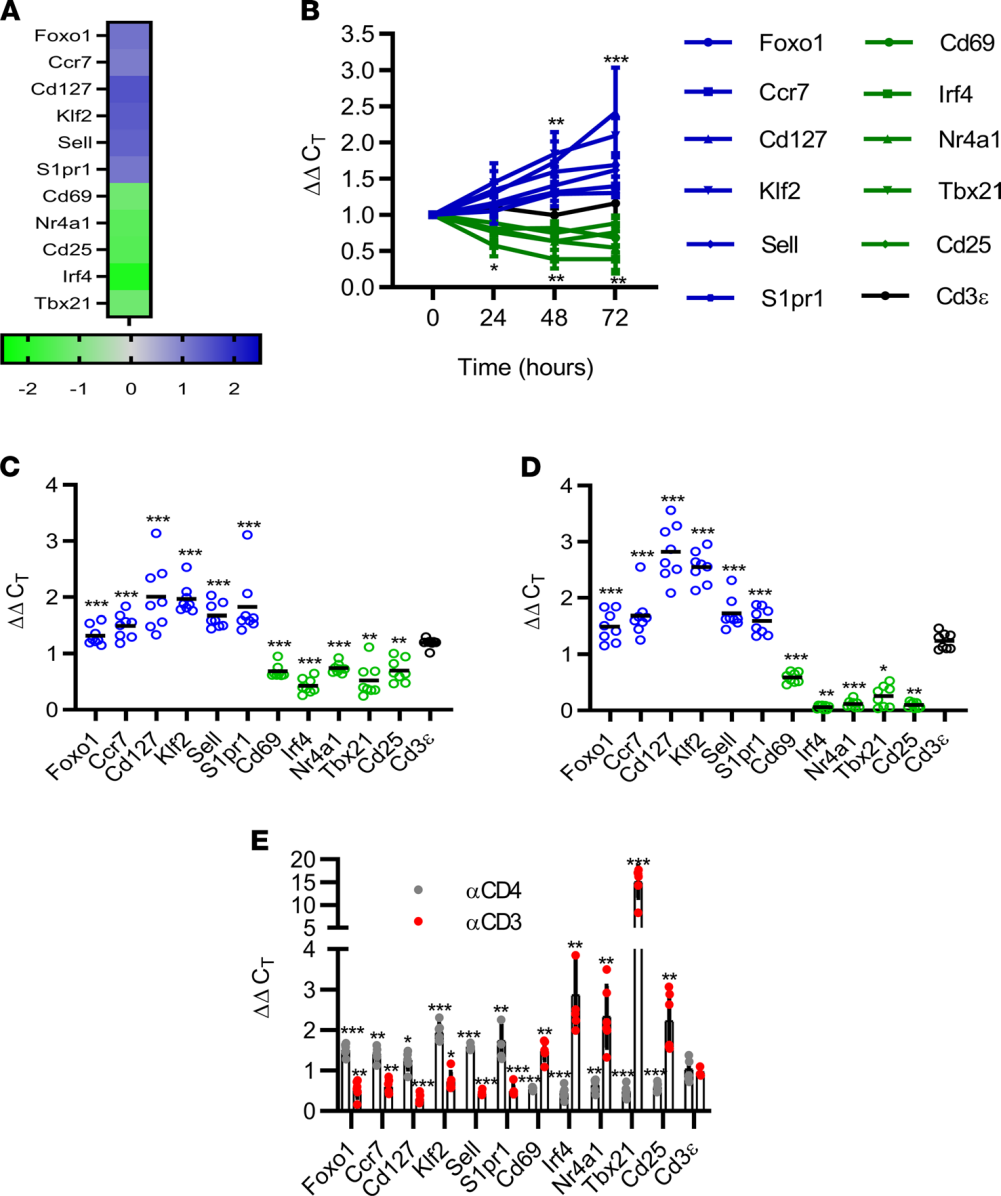
ND αCD4/αCD8α Abs increase Foxo1-regulated gene expression in T cells. (**A**) Microarray analysis of FACS-isolated PLN CD4^+^ T cells from BDC mice 48 hours after ND αCD4 or control Ab treatment. Values are relative to isotype control Ab–treated mice. (**B**–**E**) mRNA expression (Foxo1 axis genes in blue, activation genes in green) via qRT-PCR in (**B**) PLN CD4^+^ T cells from BDC mice (*n* = 8) after ND αCD4 (ΔΔC_T_ calculated relative to 0-hour control CD4^+^ T cells); pancreatic (**C**) CD4^+^ and (**D**) CD8^+^ T cells sorted from ND Ab–treated BDC and 8.3 mice 24 hours after treatment (*n* = 8), respectively (ΔΔC_T_ calculated relative to isotype control Ab–treated T cells); and (**E**) PLN CD4^+^ T cells from BDC mice (*n* = 6) 48 hours after treatment with ND αCD4, αCD3, or control Ab (ΔΔC_T_ calculated relative to control Ab–treated CD4^+^ T cells). *P < 0.05, **P < 0.01, ***P < 0.001, determined by Student’s *t* test (**B**–**E**). Error bars depict SD. Data are pooled from 2 experiments.

Consistent with our prior work, tolerance induction by CoRT was linked to suppression of TCR signaling–driven events in Teffs shortly after ND αCD4/αCD8α treatment. Expression of IFN-γ, IL-2, and the proliferation marker Ki67 was reduced, coupled with egress of T cells in the pancreas and PLN but not the spleen (Supplemental Figure 1; supplemental material available online with this article; https://doi.org/10.1172/jci.insight.149130DS1) (25, 27). To define the early molecular events modulated by CoRT, we profiled the transcriptome of diabetogenic T cells. CD3^+^CD4^+^ T cells were sorted by FACS from the PLN of NOD mice expressing the IA^g7^-restricted BDC2.5 (BDC) clonotypic TCR 48 hours after treatment with ND αCD4 or isotype control Ab. Expression of genes associated with TCR signaling strength, activation, proliferation, and Teff function were reduced by ND αCD4 (Figure 2A and Supplemental Figure 2A). Strikingly, ND αCD4 increased expression of signature genes canonically regulated by the transcription factor Foxo1. Expression of *Foxo1* as well as downstream targets *Klf2*, *Cd127*, *Ccr7*, *Sell*, and *S1pr1* was elevated in BDC CD4^+^ T cells bound by ND αCD4 (Figure 2A). BDC CD4^+^ T cells isolated from the PLN of ND αCD4–treated BDC mice exhibited a dynamic increase in expression of Foxo1 axis–related genes that was concomitant with decreased expression of genes regulated by TCR signaling (*Nr4a1*, *Cd25*, *Cd69*, *Tbx21*) (Figure 2B). Expression of *Cd3*ε, and genes encoding costimulatory molecules such as CD28, CD40L, and CTLA-4, however, were unaffected by ND αCD4, indicating only select genes associated with TCR signaling were altered after CoRT (Figure 2B). A similar transcriptional profile was detected in pancreas-infiltrating CD4^+^ and CD8^+^ T cells sorted via FACS from (i) ND αCD4–treated BDC mice (Figure 2C) and (ii) ND αCD8α–treated NOD mice expressing the H2K^d^-restricted 8.3 clonotypic TCR (hereafter referred to as “8.3 mice”) (Figure 2D). Importantly, analogous transcriptional profiles were also detected in polyclonal CD4^+^ and CD8^+^ T cells sorted from the pancreas of 16-week-old NOD female mice treated with both ND αCD4 and ND αCD8α (Supplemental Figure 2, B and C). Treatment of BDC mice with αCD3 Ab (clone 2C11), a therapy shown to reverse diabetes in NOD mice, however, decreased expression of Foxo1-axis genes but increased expression of TCR-driven genes (*Irf4*, *Nr4a1*, *Cd25*, *Cd69*, *Tbx21*) in PLN BDC CD4*^+^* T cells (Figure 2E). This finding is consistent with a negative regulatory effect of TCR signaling on Foxo1 expression and transcription activity (31, 32).

**Figure 3:**
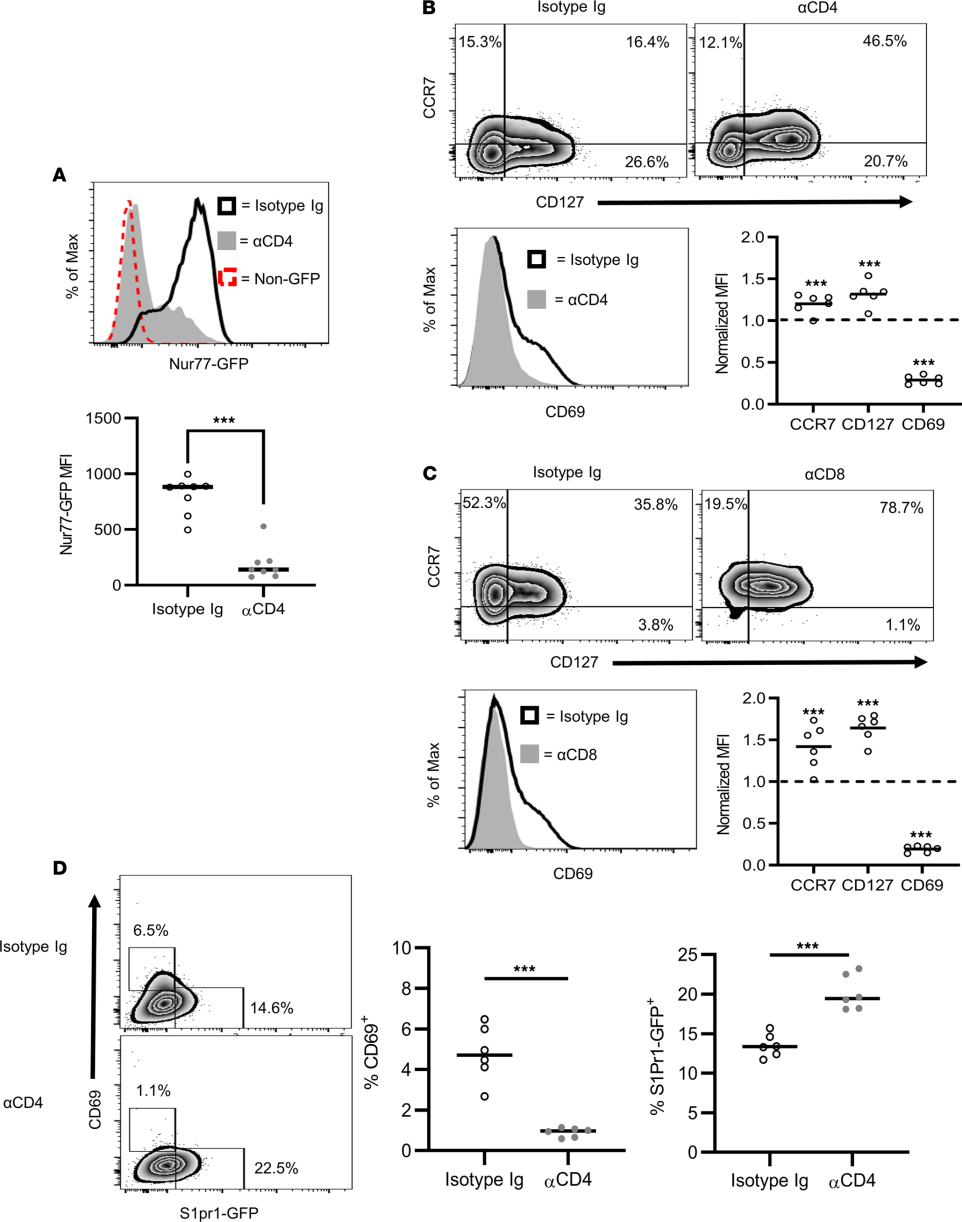
ND αCD4/αCD8α Abs inhibit TCR signaling regulating Foxo1-mediated gene expression in T cells. (**A**) BDC.Nur77-GFP mice (*n* = 6) were treated with ND αCD4 or control Ab, and 72 hours later pancreatic T cells were analyzed by FACS. (**B**) BDC and (**C**) 8.3 mice were treated with ND or control Ab (*n* = 6), and 72 hours later pancreatic T cells were analyzed by FACS. MFI normalized to control Ab. (**D**) BDC.S1pr1-GFP mice were treated with ND αCD4 or control Ab (*n* = 6), and 72 hours later pancreatic T cells were analyzed by FACS. ***P < 0.001, determined by Student’s *t* test (**A**–**D**). Error bars depict SD. Data are pooled from 2 experiments.

Paralleling our transcriptional profiling, TCR activity, measured by intracellular Nur77 expression, was reduced by ND αCD4 versus isotype control Ab in CD4^+^ Teffs residing in the pancreas of BDC mice expressing a Nur77-GFP reporter cassette (BDC.Nur77-GFP) (Figure 3A). In addition, surface expression of the Foxo1-regulated gene products CD127 and CCR7 was increased on pancreas-resident CD4^+^ and CD8^+^ T cells from ND αCD4– and ND αCD8α–treated BDC and 8.3 mice, respectively (Figure 3, B and C). In contrast, CD69 surface expression was downregulated by CoRT (Figure 3, B and C), consistent with diminished *Cd69* mRNA levels (Figure 2, A–D). Similar increases in CD127 and CCR7 were detected on pancreatic CD4^+^ and CD8^+^ T cells from ND αCD4/αCD8α–treated NOD mice (Supplemental Figure 2D). Notably, the frequency of pancreas-resident S1PR1-expressing CD4^+^ T cells was also increased in ND αCD4–treated BDC mice expressing an S1PR1-GFP reporter cassette (Figure 3D). The Foxo1/KLF2/S1PR1 axis is known to establish a circulatory phenotype in T cells, and simultaneously suppress tissue-resident memory (Trm) development (40). Indeed, ND αCD4 drove rapid upregulation of transcripts characteristic of recirculating T cells and diminished expression of key tissue residency genes (Supplemental Figure 2E) (41). Furthermore, the frequency of pancreatic CD103-expressing CD8^+^ T cells was reduced by ND αCD4/αCD8α treatment (Supplemental Figure 2F); CD103 is a key marker for CD8^+^ Trm cells. Overall, these findings demonstrate that CoRT induces marked changes in the Foxo1 transcriptional pathway. Furthermore, CoRT promoted a shift in gene expression in pancreatic tissue–resident T cells toward a recirculating phenotype.

### The level of TCR signaling impacts CoRT-induced regulation of the Foxo1/S1pr1 transcription axis.

We have previously shown that TCR signaling is required for islet T cell residency; treatment of NOD mice with the calcineurin inhibitor FK506 induces egress of islet-resident T cells (27). Foxo1 transcriptional activity is repressed by TCR signaling through activation of AKT, which phosphorylates and promotes Foxo1 translocation to the cytoplasm (31, 32). Since CoRT dampens TCR signaling (Figure 3A), we hypothesized that the magnitude of antigen stimulation is important for CoRT-induced effects on Foxo1 activity and Teff properties and function (27, 42, 43).

**Figure 4:**
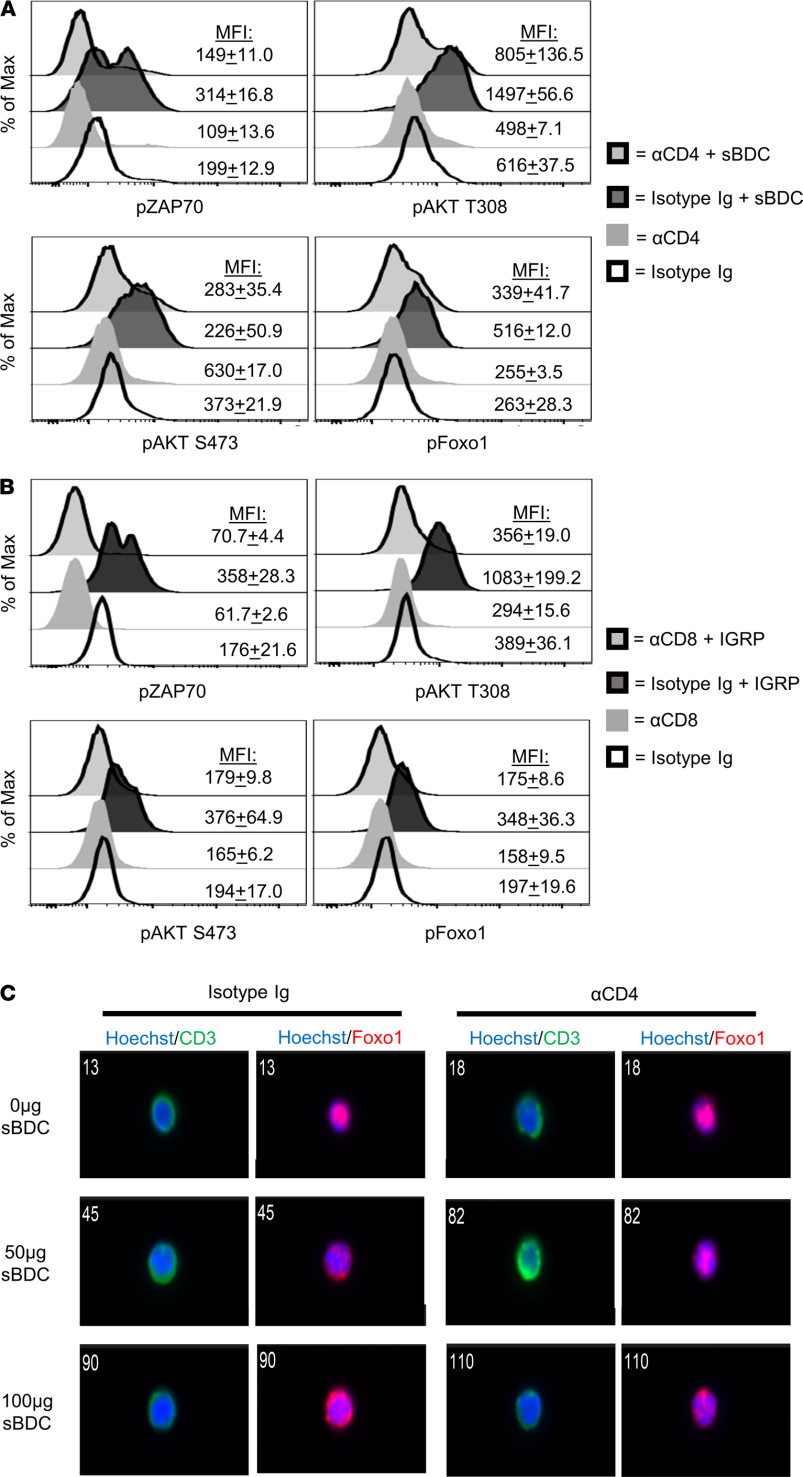
CoRT promotes nuclear residency of Foxo1. (**A**) BDC and (**B**) 8.3 mice were treated with ND αCD4 or αCD8α, respectively, or control Ab; 24 hours later, splenocytes were isolated and stimulated in vitro for 30 minutes (p-ZAP70) or 1 hour (p-AKT/Foxo1) with 1 μg cognate peptide (*n* = 6), and TCR signaling was assessed by FACS. (**C**) BDC mice were treated with ND αCD4 or control Ab (*n* = 6), immunized 18 hours later with sBDC, and 1 hour after that PLN T cells were examined by ImageStream flow cytometry at ×60 magnification. Data are pooled from 2 to 3 experiments.

Initially, we assessed the effects of CoRT on antigen-stimulated phosphorylation of ZAP70, AKT, and Foxo1. BDC and 8.3 mice were treated with ND αCD4 or ND αCD8α, respectively, or isotype control Ab, and splenic T cells were stimulated ex vivo with cognate peptide. ND αCD4 reduced the level (e.g., mean fluorescence intensity [MFI]) of activated phosphorylated ZAP70 (p-ZAP70) and p-AKT in BDC CD4^+^ T cells following stimulation with sBDC, a mimetic peptide (Figure 4A and Supplemental Figure 3A). Notably, the level of inactivated p-Foxo1 was also significantly reduced by ND αCD4 (Figure 4A and Supplemental Figure 3A). Similar, but more robust effects were seen with ND αCD8α–bound 8.3 CD8^+^ T cells stimulated with IGRP_206–214_, which exhibited strikingly lower levels of p-ZAP70 and p-AKT (Figure 4B and Supplemental Figure 3B).

Consistent with the above results, CoRT effects on Foxo1 nuclear localization were impacted by the strength of antigen stimulation in vivo. BDC mice were treated with ND αCD4 or isotype control Ab for 18 hours, injected with sBDC, and 1 hour later PLN T cells were isolated and examined by ImageStream flow cytometry. In isotype control Ab–treated BDC mice injected with 50 μg sBDC, BDC CD4^+^ T cells exhibited distinct Foxo1 cytoplasmic localization, quantitated as a reduced similarity index (SI) compared with the no-peptide, isotype control Ab cohort (Figure 4C and Supplemental Figure 3C). In contrast, Foxo1 was predominantly localized to the nucleus in BDC CD4^+^ T cells from ND αCD4–treated BDC mice injected with 50 μg sBDC; no significant change in SI relative to the no-peptide, ND αCD4–treated group of animals was observed (Figure 4C and Supplemental Figure 3C). Conversely, an increased sBDC dose (100 μg) resulted in significant cytoplasmic Foxo1 localization in ND αCD4–bound BDC CD4^+^ T cells, and a reduced SI relative to the no-peptide, ND αCD4–treated cohort (Figure 4C and Supplemental Figure 3C).

**Figure 5:**
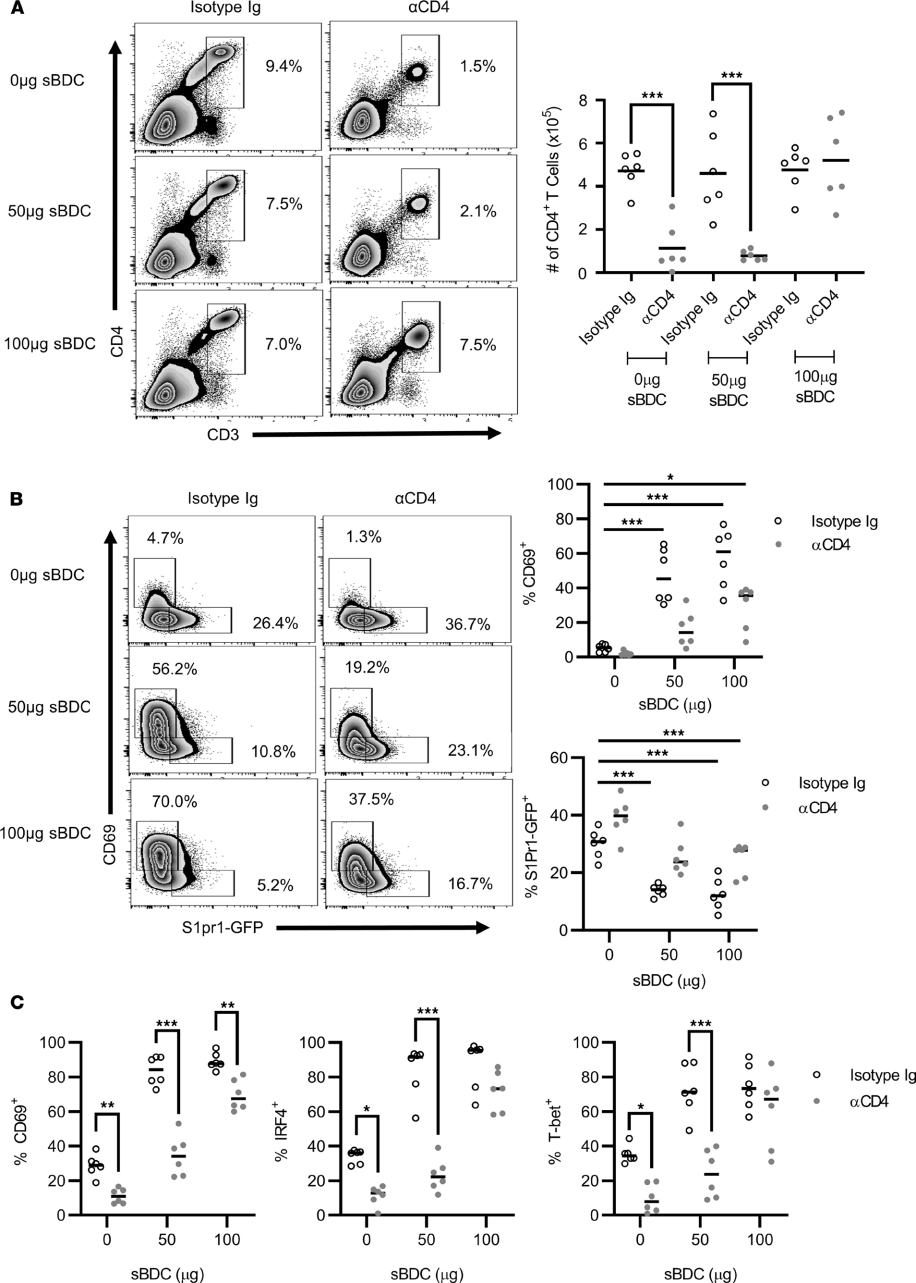
TCR signaling–dependent pancreatic T cell tissue residency is lost upon CoRT-induced Foxo1/S1PR1. (**A** and **B**) BDC and (**C**) BDC.S1pr1-GFP mice were treated with ND αCD4 or control Ab (*n* = 6), immunized 6 hours later with sBDC, and after 18 hours pancreatic CD4^+^ T cells were examined by FACS. Error bars depict SD. *P < 0.05, **P < 0.01, ***P < 0.001, determined by 2-way ANOVA (**A**–**C**). Data are pooled from 2–3 experiments.

The level of antigen stimulation also affected CoRT-induced T cell egress and suppression of effector function. The majority of pancreatic BDC CD4^+^ T cells underwent egress in animals treated with ND αCD4 alone or ND αCD4 plus 50 μg sBDC (Figure 5A). The latter correlated with an increased frequency of S1PR1-GFP^+^ BDC CD4^+^ T cells in analogously treated BDC.S1pr1-GFP mice (Figure 5B). Molecules associated with TCR signaling strength, activation, effector function, and tissue retention were also reduced in the ND αCD4 plus 50 μg sBDC cohort (Figure 5C and Supplemental Figure 3D). In contrast, no pancreatic egress of ND αCD4–bound BDC CD4^+^ T cells was seen with an increased sBDC dose (100 μg) (Figure 5A), which coincided with a decreased percentage of S1PR1-GFP^+^ cells (Figure 5B) and an increased frequency of IRF4^+^, CD69^+^, and T-bet^+^ pancreatic T cells (Figure 5C and Supplemental Figure 3D). Taken together, these results demonstrate that CoRT reduces TCR signaling in a graded fashion, thereby regulating the Foxo1/S1PR1 transcriptional axis.

### CoRT induces S1P-dependent T cell egress from the pancreas.

**Figure 6:**
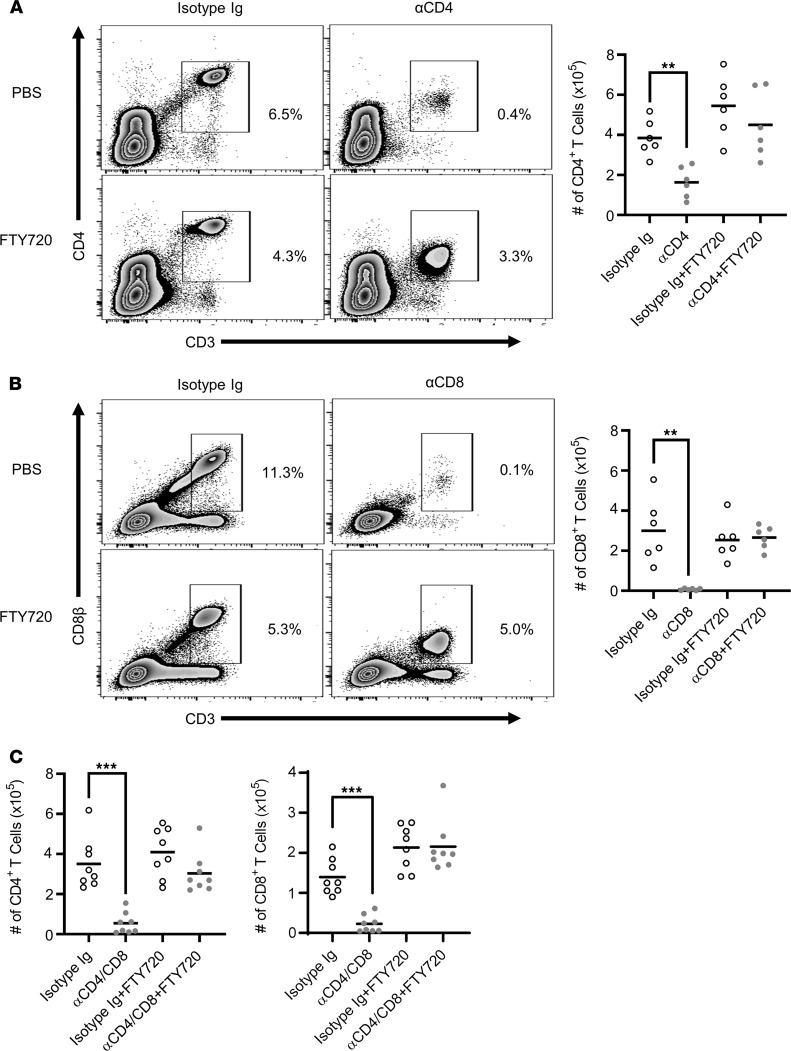
CoRT induces S1P-dependent T cell egress from the pancreas and PLN. (**A**) BDC, (**B**) 8.3, and (**C**) NOD mice were treated with 1 mg/kg FTY720, and 24 hours later received ND αCD4 or αCD8α, or both ND αCD4 and αCD8α, or control Ab plus daily injections of FTY720 for 72 hours (*n* = 6–8). Pancreatic CD4^+^ and CD8^+^ T cells were enumerated by FACS. Data are pooled from 2 experiments. Error bars depict SD. **P < 0.01, ***P < 0.001, determined by 2-way ANOVA (**A**–**C**).

The above changes in S1PR1 expression and islet T cell residency induced by ND αCD4/αCD8α binding suggested that CoRT-induced egress of pancreatic T cells was regulated by S1P. To test this scenario, we used the S1PR family–specific inhibitor FTY720 (44). As expected, ND αCD4 and ND αCD8α treatment of BDC and 8.3 mice, respectively, reduced pancreatic CD4^+^ and CD8^+^ T cell numbers relative to isotype control Ab–treated animals (Figure 6, A and B). In contrast, no change in pancreatic T cell numbers was observed in BDC and 8.3 mice cotreated with FTY720 plus ND αCD4 or ND αCD8α, respectively (Figure 6, A and B). Treatment of NOD mice with ND αCD4/αCD8α plus FTY720 similarly blocked egress of CD4^+^ and CD8^+^ T cells from the pancreas (Figure 6C). These findings demonstrate that ND αCD4/αCD8α–induced T cell egress of inflamed tissues is mediated in an S1P-dependent manner, correlating with increased Foxo1-dependent T cell expression of S1PR1.

### The efficiency of CoRT-driven suppression and egress of Teffs is Foxo1 dependent.

**Figure 7:**
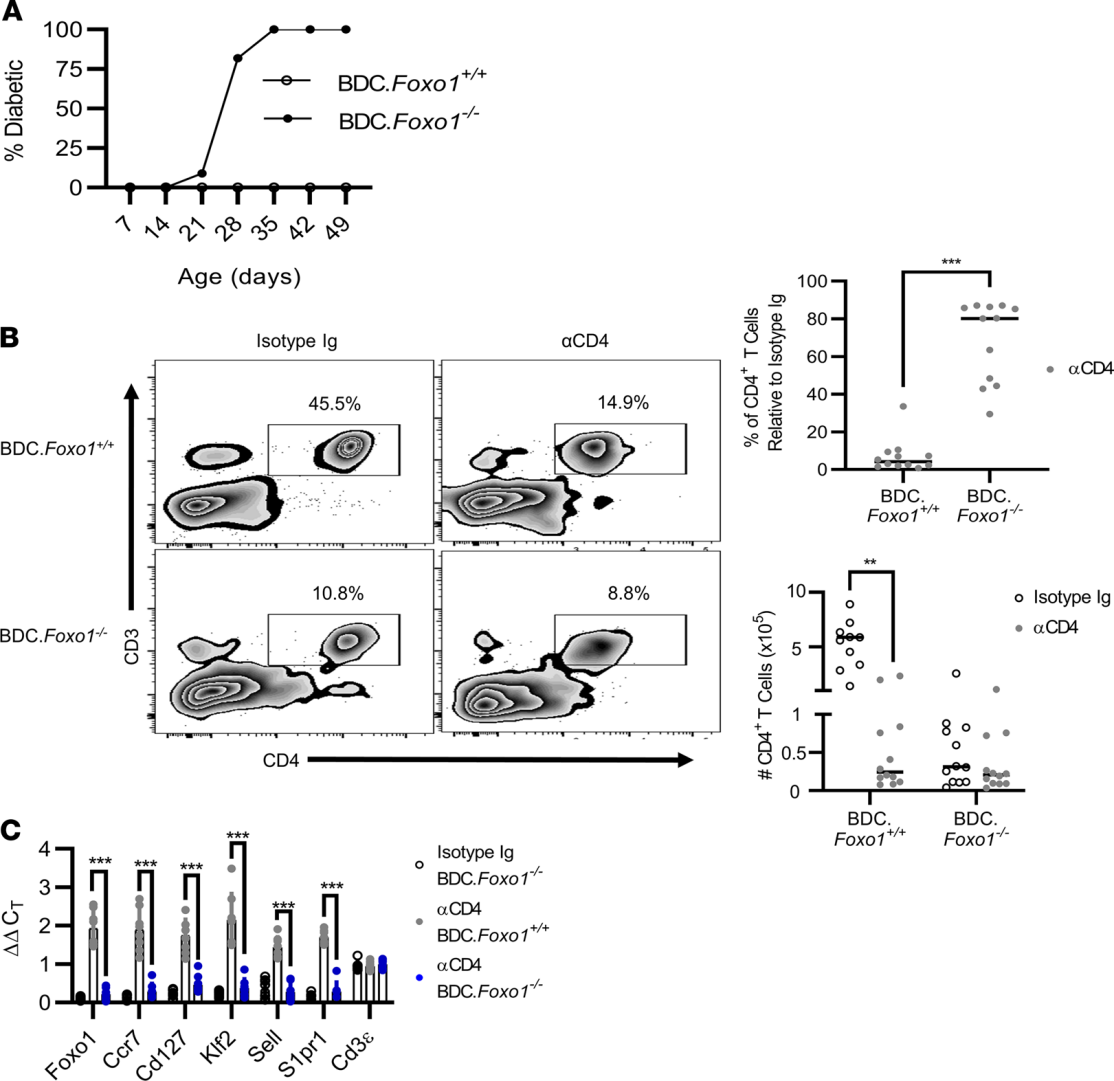
CoRT-induced T cell egress is Foxo1 dependent. (**A**) Diabetes incidence in BDC.*Foxo1^+/+^* and BDC.*Foxo1^–/–^* female mice (*n* = 12). (**B**) BDC.*Foxo1^+/+^* and BDC.*Foxo1^–/–^* mice were treated with ND αCD4 or control Ab (*n* = 8), and 72 hours later pancreatic CD4^+^ T cells were enumerated by FACS. (**C**) mRNA was measured in splenic CD4^+^ T cells purified from BDC.*Foxo1^+/+^* and BDC.*Foxo1^–/–^* mice (*n* = 8) 72 hours after ND αCD4 or control Ab treatment (ΔΔC_T_ calculated versus control Ab). Error bars depict SD. **P < 0.01, ***P < 0.001, determined by Student’s *t* test (**B**) or 2-way ANOVA (**C**). Data are pooled from 3–4 experiments.

To further establish the relative contribution of Foxo1 in CoRT-induced T cell egress and suppression of effector function, we generated NOD BDC.*Foxo1^fl/fl^.Lck^Cre^* (BDC.*Foxo1^–/–^*) mice that lack Foxo1 expression specifically in T cells (Supplemental Figure 4A). BDC.*Foxo1^–/–^* mice exhibited an overall decrease in peripheral T cells, but an increased frequency of Teffs relative to Foxo1-replete BDC.*Foxo1^fl/fl^* (BDC.*Foxo1^+/+^*) mice (Supplemental Figure 4B). This increase in Teffs is consistent with results obtained with *Foxo1^fl/fl^.Lck^Cre^* mice on the C57BL/6 genetic background (28). The Teff pool of BDC.*Foxo1^–/–^* mice was marked by an elevated percentage of CD44^+^ T cells expressing IFN-γ coupled with TNF-α, IL-17, and IL-2 (Supplemental Figure 4C). Strikingly, all BDC.*Foxo1^–/–^* female mice (*n* = 12) developed diabetes between 3 and 5 weeks of age (Figure 7A), which was marked by islet infiltration (Supplemental Figure 4D), suggesting a prominent role for Foxo1 in T1D development. Despite the rapid development of overt diabetes, the number of splenic, PLN, and pancreas-infiltrating CD4^+^ T cells was reduced in BDC.*Foxo1^–/–^* mice approximately 10-fold compared with BDC.*Foxo1^+/+^* mice, which is consistent with reduced peripheral T cell numbers in Foxo1-deficent mice (Supplemental Figure 4, E–G) (29).

The effect of Foxo1 deficiency on CoRT-induced T cell egress was assessed. As expected in BDC.*Foxo1^+/+^* mice treated with ND αCD4, pancreatic T cell numbers were reduced up to 10-fold versus the isotype control Ab group (Figure 7B). In contrast, Foxo1 deficiency blocked CoRT-induced T cell egress, as ND αCD4 had no significant effect on pancreatic T cell numbers in BDC.*Foxo1^–/–^* mice versus the isotype control Ab cohort (Figure 7B). Indeed, the efficiency of ND αCD4–induced pancreatic T cell egress differed significantly between BDC.*Foxo1^–/–^* and BDC.*Foxo1^+/+^* mice. In ND αCD4–treated BDC.*Foxo1^+/+^* mice, greater than 90% of BDC CD4^+^ T cells were purged from the pancreas after 72 hours (Figure 7B). In contrast, the pancreatic BDC CD4^+^ T cell pool was only reduced by approximately 40% in ND αCD4–treated BDC.*Foxo1^–/–^* mice (Figure 7B). T cell egress was readily induced by ND αCD4 in the pancreas of NOD.*Lck^Cre^* mice (Supplemental Figure 4H), indicating that limited T cell egress in BDC.*Foxo1^–/–^* mice was due to *Foxo1* deletion. As expected, ND αCD4 failed to upregulate mRNA expression for Foxo1-axis genes, such as *S1pr1* in T cells isolated from the pancreas of BDC.*Foxo1^–/–^* mice (Figure 7C).

**Figure 8:**
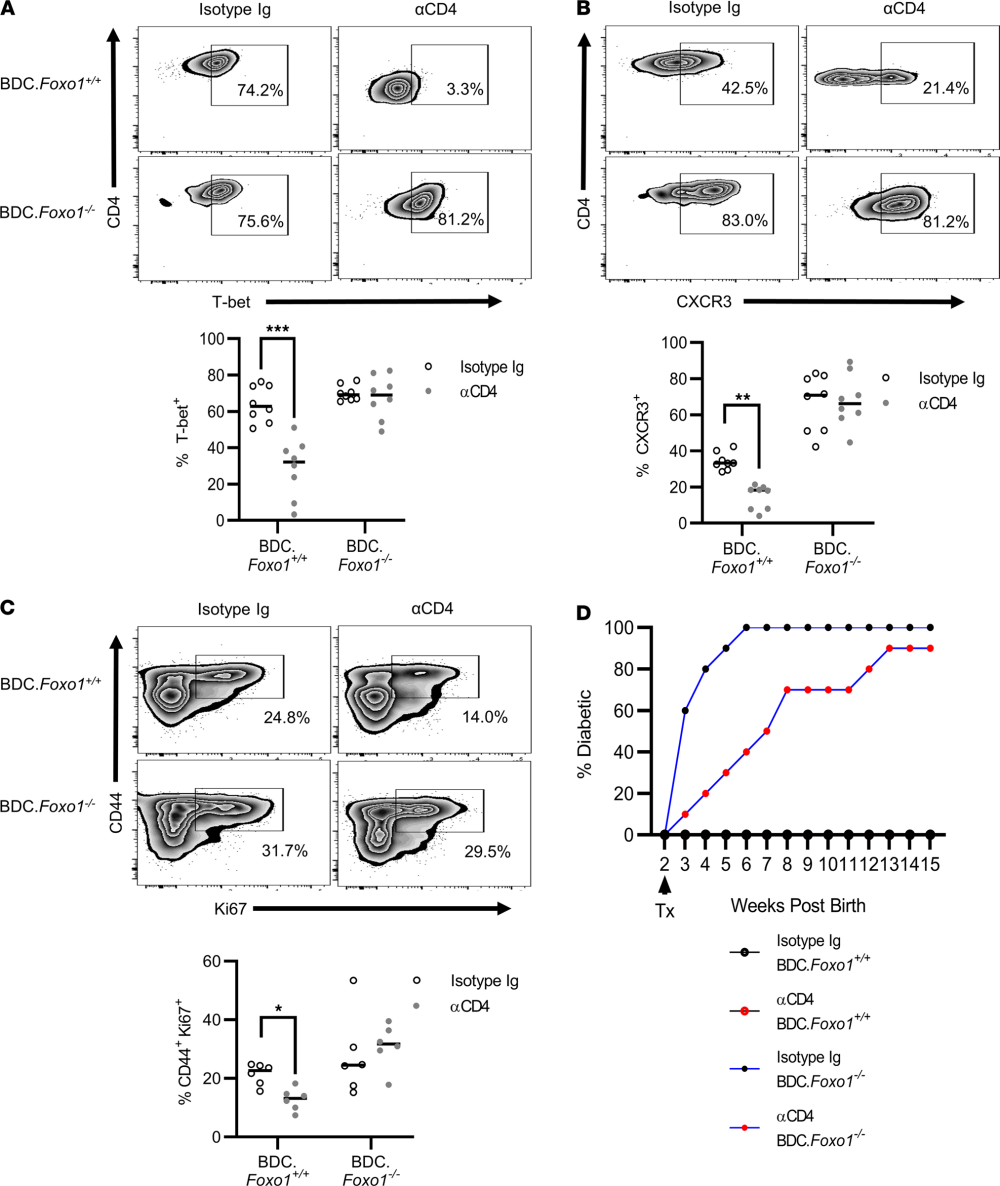
CoRT-mediated inhibition of Teff function is Foxo1 dependent. (**A**–**C**) BDC.*Foxo1^+/+^* and BDC.*Foxo1^–/–^* mice were treated with ND αCD4 or control Ab (*n* = 6), and FACS analyses were carried out (**A** and **B**) 24 hours later on pancreatic T cells, and (**C**) 48 hours later on PLN T cells. (**D**) Female BDC.*Foxo1^+/+^* and BDC.*Foxo1^–/–^* mice (*n* = 10) were treated with ND αCD4 or control Ab at 2 weeks of age and diabetes incidence was monitored. Error bars depict SD. *P < 0.05, **P < 0.01, ***P < 0.001, determined by 2-way ANOVA (**A**–**C**). Data are pooled from 3–4 experiments.

Expression of the Th1-defining lineage factors T-bet and CXCR3 was a reduced in BDC.*Foxo1^+/+^* mice but maintained in BDC.*Foxo1^–/–^* mice after ND αCD4 (Figure 8, A and B). Furthermore, T cell proliferation in the PLN, as measured by Ki67, was dampened in ND αCD4–treated BDC.*Foxo1^+/+^* mice, whereas BDC.*Foxo1^–/–^* T cell proliferation persisted after CoRT (Figure 8C). Interestingly, ND αCD4 only delayed the development of overt diabetes in BDC.*Foxo1^–/–^* mice (Figure 8D). Together, these results demonstrate that Foxo1 regulates (i) the progression of T1D, (ii) the efficiency of CoRT-induced T cell egress via S1PR1, and (iii) suppression of type 1 effector function.

**Figure 9:**
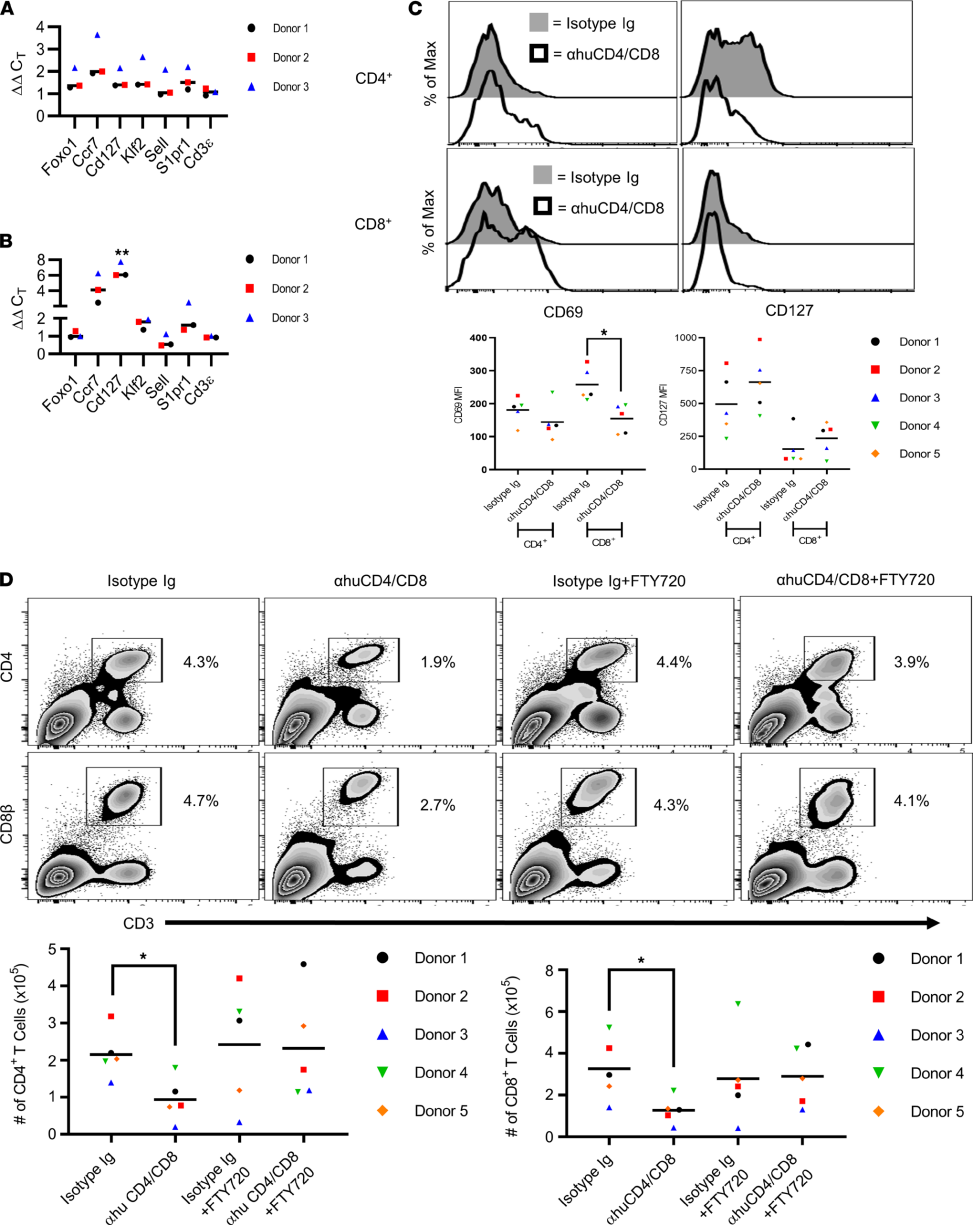
ND αhuCD4/αhuCD8α Abs promote S1P-dependent human T cell egress during xGvHD. (**A**–**C**) NRG.PBL mice (*n* = 3–5 human donors; each symbol is the average for the cohort of a given donor) were treated with ND αhuCD4/αhuCD8α or control Ab. (**A** and **B**) mRNA measured in purified human (**A**) CD4^+^ and (**B**) CD8^+^ T cells (ΔΔC_T_ calculated relative to control). (**C**) Pancreatic human CD4^+^ and CD8^+^ T cells examined by FACS. (**D**) NRG.PBL mice were pretreated with 1 mg/kg FTY720 for 24 hours, and then received 1 injection of ND αhuCD4 and αhuCD8α or control Ab, plus daily injections of FTY20 for 72 hours (*n* = 5 human donors; each symbol is the average for the cohort of a given donor). Pancreatic human CD4^+^ and CD8^+^ T cells were then enumerated by FACS. Error bars depict SD. *P < 0.05, **P < 0.01, determined by Student’s *t* test (**A**–**C**) or 2-way ANOVA (**D**). Data are pooled from 3–5 experiments.

*ND* α*huCD4/*α*huCD8 upregulates the Foxo1 transcriptional axis and promotes S1P-dependent human T cell egress of inflamed tissue*. We had shown earlier that ND human (hu) IgG4 specific for huCD4 (clone CH5g5) and huCD8α (clone CH9d2) induces egress of pathogenic CD4^+^ and CD8^+^ T cells from inflamed tissues in NRG.PBL humanized mice, which develop xenogeneic graft-versus-host disease (xGVHD) (27). This humanized mouse model permits analyses of tissue-resident, antigen-stimulated human CD4^+^ and CD8^+^ Teffs. Accordingly, we assessed whether ND αhuCD4/αhuCD8α binding modulates the Foxo1 transcriptional axis. Cohorts of NRG.PBL mice, established with PBMCs from 3 individual donors, were treated with ND αhuCD4/αhuCD8α or isotype control Ab, and splenic huCD4^+^ and huCD8^+^ T cells were isolated. Expression levels of Foxo1-regulated genes, including *S1PR1*, tended to increase after ND αhuCD4/αhuCD8α treatment compared with the isotype control Ab cohort (Figure 9, A and B). Furthermore, ND αhuCD4/αhuCD8α therapy increased CD127 MFI and decreased CD69 MFI on huCD4^+^ and huCD8^+^ T cells (Figure 9C).

Elevated *S1PR1* expression suggested that CoRT-induced tissue egress of huCD4^+^ and huCD8^+^ Teffs was also S1P dependent. To test this, cohorts of NRG.PBL mice, established from 5 individual donors, were treated with ND αhuCD4/αhuCD8α or isotype control Ab plus/minus FTY720. As expected, ND αhuCD4/αhuCD8α alone significantly reduced the number of huCD4^+^ and huCD8^+^ T cells in the pancreas 72 hours after treatment (Figure 9D). In contrast, FTY720 cotreatment blocked pancreatic T cell egress induced by ND αhuCD4/αhuCD8α (Figure 9D). Together, these findings indicate that, similarly to murine T cells, CoRT induces S1P-dependent egress that correlates with upregulation of the Foxo1 transcriptional axis in huCD4^+^ and huCD8^+^ Teffs.

### CoRT-induced prevention of T1D and long-term tolerance is due to establishment of exhausted pancreas-resident CD8^+^ Teffs that reseed the pancreas.

**Figure 10:**
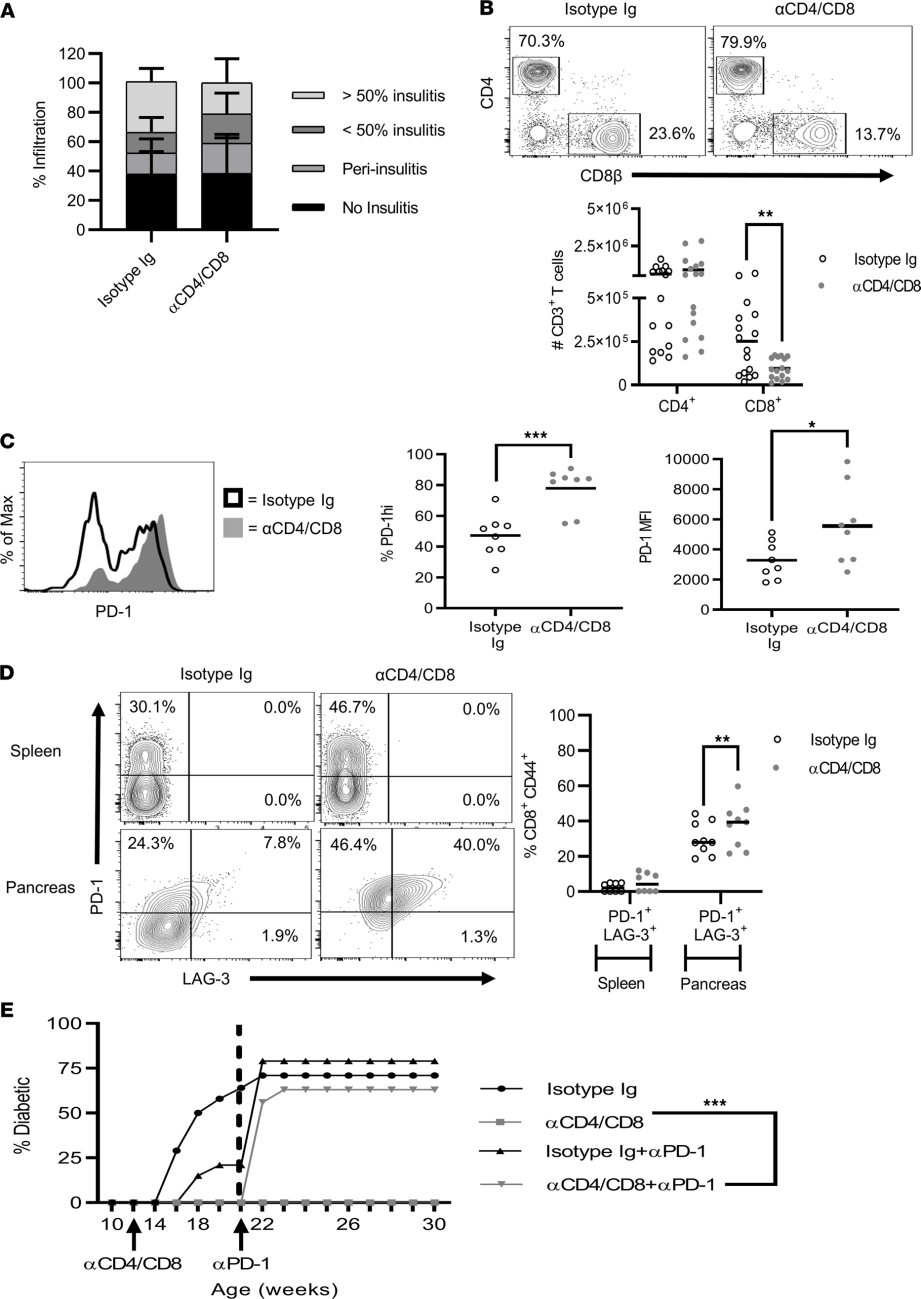
ND αCD4/αCD8 treatment promotes CD8^+^ Teff exhaustion. (**A**–**E**) Twelve-week-old NOD female mice were treated with 2 injections 1 day apart with ND αCD4/αCD8α or control Ab. (**A**) Insulitis scored by immunohistochemistry 60 days after ND αCD4/αCD8α (*n* = 10) and control Ab (*n* = 10). (**B**) Pancreatic CD4^+^ and CD8^+^ T cell numbers were determined via FACS 60 days after treatment with ND αCD4/αCD8α (*n* = 16) or control Ab (*n* = 16). (**C**) Pancreatic and splenic CD44^+^ CD8^+^ Teffs were examined by FACS for PD-1 and LAG-3 expression 60 days after treatment with ND αCD4/αCD8α (*n* = 8) or control Ab (*n* = 8). (**D**) Twelve-week-old NOD female mice were treated with ND αCD4/αCD8α (*n* = 15) and control Ab (*n* = 14) alone, or 60 days later also received αPD-1 Ab (CoRT plus αPD-1 [*n* = 16]; isotype control Ab plus αPD-1 Ab [*n* = 14]), and diabetes onset was monitored. *P < 0.05, **P < 0.01, ***P < 0.001, determined by Student’s *t* test (**A**–**D**) or Kaplan-Meier (**E**). Error bars depict SD. Data are pooled from 2 or more experiments.

CoRT effectively reestablished long-term self-tolerance in NOD mice at both clinical and late preclinical T1D stages (Figure 1, A, B, and D). Analogous with our earlier findings, persistent remission induced by CoRT in diabetic NOD mice was marked by the majority of islets (>80%) remaining free of insulitis up to 200 days after ND αCD4/αCD8α treatment (Figure 1C). Therefore, it was surprising that substantial insulitis was detected in the pancreas 60 days after ND αCD4/αCD8α treatment of 12-week-old female NOD mice (Figure 10A). The frequency of insulitis was similar between the CoRT versus control cohorts, although islets exhibiting greater than 50% insulitis (21.1% versus 34.5%) were reduced, whereas peri-insulitis (20.6% versus 14.2%) was increased (Figure 10A). Furthermore, the number of pancreatic CD4^+^ T cells was similar between ND αCD4/αCD8α and the control group 60 days after treatment (Figure 10B). In contrast, pancreatic CD8^+^ T cells were significantly reduced in the ND αCD4/αCD8α cohort (Figure 10B). The frequency of pancreatic CD44^+^CD4^+^ Teffs was similar between the treatment groups, whereas a trend toward reduced pancreatic CD44^+^CD8^+^ Teffs was seen in the CoRT cohort (Supplemental Figure 6A). No significant difference was seen among the cohorts in the frequency of pancreatic FoxP3-expressing CD4^+^ T cells (Supplemental Figure 6B). These results indicate that after CoRT-induced T cell purging, the pancreas is reseeded overtime with CD4^+^ Teffs and Foxp3^+^ Tregs and exhibits a reduced CD8^+^ Teff pool.

Since NOD mice remained free of diabetes in the long term (Figure 1C) despite Teffs reseeding the pancreas after CoRT (Figure 10, A and B), we hypothesized that the pathogenicity of islet Teffs was intrinsically impaired. Notably, T cells in vivo are no longer bound by ND αCD4 and αCD8α 4 weeks after treatment (27). Initially, pancreatic T cells were examined for expression of PD-1, a signature marker for T cell exhaustion, 60 days after Ab treatment. Pancreatic CD44^+^CD4^+^ Teffs showed no marked change in the frequency of PD-1 expression between the CoRT and control Ab groups (Supplemental Figure 6C). On the other hand, pancreatic CD44^+^CD8^+^ Teffs exhibited an increased frequency and elevated surface expression of PD-1 in ND αCD4/αCD8α versus isotype control Ab mice (Figure 10C). Furthermore, the frequency of pancreatic CD44^+^CD8^+^ Teffs coexpressing PD-1 with other inhibitory molecules associated with T cell exhaustion such as LAG-3 and TIM-3 was also increased in the cohort (Figure 10D and Supplemental Figure 6E). No marked increase in PD-1^+^LAG-3^+^CD44^+^CD8^+^ and PD-1^+^TIM-3^+^CD44^+^CD8^+^ Teffs was detected in the spleen of the ND αCD4/αCD8α group. Splenic and pancreatic CD44^+^CD4^+^ Teffs showed relatively no change in coexpression of PD-1 with LAG-3 or TIM-3 in the long term after CoRT (Supplemental Figure 6D). Moreover, the expression of the transcription factors TOX (thymocyte selection–associated high-mobility group box) and Eomes, which drive T cell exhaustion, was increased within the pool of CD8^+^ Teffs infiltrating the pancreas of ND αCD4/αCD8α versus isotype Ab control mice (Supplemental Figure 6, F and G).

To determine if T cell exhaustion was playing a functional role in CoRT-mediated protection, 12-week-old NOD female mice were treated with ND αCD4/αCD8α or isotype control Ab, and then received blocking αPD-1 Ab 60 days later. As expected, all of the ND αCD4/αCD8α–only–treated NOD mice remained free of diabetes (0/11; Figure 10E). In contrast, diabetes was rapidly induced in the majority (7/12; 64%) of the ND αCD4/αCD8α cohort injected with αPD-1 (Figure 10E). Taken together, these findings demonstrate that long-term protection induced by CoRT at a late preclinical T1D stage is due to a reduced pool of pancreatic CD8^+^ Teffs that are functionally exhausted.

## Discussion

We and others have reported that CoRT is a robust approach to reestablish self-tolerance in models of T cell–mediated autoimmunity, as well as promote transplantation tolerance (25, 27, 45–50). To effectively apply CoRT to the clinic, a detailed understanding of the conditions used for and mechanisms by which CoRT mediates T cell tolerance is essential. The current study demonstrates that CoRT at both late-preclinical and clinical T1D stages is effective in establishing long-term self-tolerance. Furthermore, early events induced by CoRT that regulate Teff function and tissue retention are underpinned by activation of the Foxo1 transcriptional axis. Moreover, CoRT has persistent effects that intrinsically block the pathogenicity of islet-resident Teffs that reseed the pancreas over time. Our findings underscore the complexity of the tolerogenic events induced by CoRT through modulating CD4 and CD8 function.

The attenuation of TCR signaling is central to the tolerogenic effects of CoRT (Figures 2–5) (25, 27). TCR stimulation plays a dynamic role in the function and maintenance of tissue-infiltrating autoreactive T cells (Figures 2 and 3) (51, 52). Our data show that CoRT suppressed transcription needed for Teff division, proinflammatory function, and tissue residency through the tuning of TCR signaling. Diminished TCR signaling correlated with ND αCD4/αCD8α–mediated internalization of CD4 and CD8 and reduction of LCK, which is needed for activation of ZAP70 (Supplemental Figure 5, A and B). The requirement for CD4 and CD8 to contribute to TCR signaling is typically reduced as antigen stimulation is increased (53). Indeed, this was readily seen in vivo, as heightened antigen stimulation of BDC CD4^+^ T cells overcame the inhibitory effects of CoRT on TCR signaling, namely that Teff egress was blocked and Teff function was rescued (Figures 4 and 5). In contrast, ND αCD8α–treated CD8^+^ T cells showed a greater dependence on coreceptor signaling, which may be reflective of an increased need for CD8 to facilitate TCR/peptide/MHC I signaling in comparison with CD4 (53, 54). Interestingly, stimulation with PMA and ionomycin, in contrast to peptide, readily induced TCR signaling in Ab-bound T cells, further indicating that CoRT therapy inhibits proximal TCR signaling events (Supplemental Figure 1B) (27). Autoreactive T cells are generally viewed as low avidity/low affinity Teffs, and therefore are expected to be more dependent on coreceptor molecule signaling (55). This may in part explain the rapid and robust tolerogenic effects of CoRT on pancreatic Teffs (Figures 2 and 3, and Supplemental Figures 1 and 2).

A consequence of CoRT attenuation of TCR signaling was upregulation of the Foxo1 transcriptional axis in Teffs. This finding is consistent with TCR signaling negatively regulating Foxo1 activity via AKT-mediated phosphorylation (31, 32). CoRT reduced levels of p-AKT and p-Foxo1 while maintaining Foxo1 nuclear localization in peptide-stimulated T cells (Figure 4C). Results obtained with BDC.*Foxo1^–/–^* mice indicate at least 2 key non–mutually exclusive roles for Foxo1 in CoRT-mediated T cell egress. Firstly, Foxo1 is necessary for CoRT-induced T cell egress. The efficiency of pancreatic T cell egress in ND αCD4–treated BDC.*Foxo1^–/–^* mice was markedly reduced, reflecting the lack of upregulation of S1PR1. Importantly, CoRT-induced egress was dependent on S1P (Figure 6), supported by our earlier results showing that PLN BDC CD4^+^ T cells from ND αCD4–treated BDC mice exhibit enhanced reactivity to S1P in vitro (26). In addition, CoRT decreases expression of CD69 that binds to and negatively regulates the function of S1PR1, which is expected to further enhance S1P-mediated chemotaxis (56). Blockade of CoRT-mediated egress was more effective in FTY720-treated BDC mice than BDC.*Foxo1^–/–^* mice, likely due to FTY720 suppression of additional S1PR family members such as S1PR3, S1PR4, and S1PR5 that respond to S1P (44). Interestingly, gene expression data revealed that CoRT induces S1PR4 expression in addition to S1PR1 (Supplemental Figure 2A). On the other hand, *Foxo1^–/–^* T cells continue to express S1PR4 (57), and we have reported that CoRT enhances T cell sensitivity to CXCL12, a CXCR4 ligand (26). CXCR4 expression is regulated by Foxo1, and was increased by ND αCD4 binding (Supplemental Figure 5C) (58).

Secondly, Foxo1 is required for CoRT-mediated suppression of Teff function. ND αCD4 failed to suppress T-bet and CXCR3 expression in BDC.*Foxo1^–/–^* mice, whereas Th1 lineage factors were suppressed by CoRT in Foxo1-replete CD4^+^ T cells (Figures 2, 3, 7, and 8). Importantly, Foxo1 has been reported to inhibit T-bet function (33, 34). Interestingly, a recent study demonstrated that TIGIT signaling enhances the function of FOXP3^+^ Tregs from patients with multiple sclerosis by upregulating Foxo1 that blocks T-bet expression and limits IFN-γ secretion (59). In T1D, IFN-γ has cytotoxic effects on β cells (60). In addition, we have shown that the type 1/Th1/Tc1 phenotype plays a key role in islet T cell residency via IFN-γ secretion, which helps drive local expression of retention cues such as CXCL9 and CXCL10 in a feed-forward loop (27). However, NOD mice lacking IFN-γ develop T1D normally, suggesting other factors may play a key role in pancreatic T cell residency (61). In addition to IFN-γ, T-bet regulates expression of other genes involved in T cell residency. For instance, T-bet regulates CXCR3 expression, which governs islet Teff trafficking and retention by binding to CXCL9 and CXCL10, and expression of both CXCR3 and T-bet is downregulated in ND αCD4–bound BDC CD4^+^ T cells (Figures 7 and 8) (62). Notably, NOD mice deficient in T-bet fail to develop T1D or significant islet infiltration, underscoring a fundamental role for T-bet in regulating T cell tissue residency (63).

The development and maintenance of tissue-resident cells during chronic autoimmunity, such as T1D, is an emerging field of study (64). Tissue-resident cells characteristically remain lodged in nonlymphoid tissues and in some cases draining lymph nodes with limited egress. The selective loss of T cells from the pancreas and accumulation of T cells in the blood observed in our study are consistent with CoRT reversing a tissue residency program through rapid Foxo1-mediated induction of a recirculating gene-expression program, ultimately contributing to efficient pancreatic T cell egress (Supplemental Figure 1) (25–27). Our findings show that CoRT downregulates expression of signature Trm genes such as *Cd69*, *Nr4a1*, *Nr4a3*, *Irf4*, and *Gzmb*, while increasing the expression of *Klf2*, *S1pr1*, *S1pr4*, *Sell*, and *Ccr7* that comprise the core gene signature of circulating T cells (Supplemental Figure 2) (41). Notably, the Foxo1/KLF/S1PR1 transcriptional axis is known to negatively regulate Trm cell development (40). Furthermore, a characteristic feature of Trm cells is constitutively elevated expression of T cell activation markers such as granzyme B, CD69, IRF4, PD-1, Tim3, and CD8α (even in the absence of persisting antigen), which were all downregulated by CoRT (Supplemental Figure 2) (65). Similarly, the frequency of pancreatic Teffs expressing CD103, a pivotal marker of T cell tissue retention, was reduced shortly after treatment (Supplemental Figure 2). Thus, CoRT disrupts multiple features associated with tissue residency, which likely contributes to the rapid and efficient egress of pancreatic Teffs.

Another important observation made in this study was the similarity in the effects of CoRT on murine and human Teffs. As previously reported, ND αhuCD4/αhuCD8α induced egress of pancreatic Teffs in NRG.PBL mice (Figure 9) (27). Notably, Teff egress (i) correlated with a trend in upregulation of *FOXO1* and *S1PR1* expression, and (ii) was S1P dependent (Figure 9). These findings indicate that altering CD4 and CD8 function has conserved effects on murine and human T cell properties, further strengthening the translational potential of CoRT.

Surprisingly, CoRT was found to have distinct effects on the progression of insulitis in NOD mice treated at diabetes onset versus a late preclinical T1D stage. Consistent with earlier findings, CoRT in diabetic NOD mice prevented subsequent long-term infiltration of T cell–purged islets (Figure 1C). This lack of insulitis is thought to be attributable to an increased Foxp3^+^ Treg pool residing in the PLN, which blocks activation and subsequent trafficking of β cell–specific T cells into the islets (25). In contrast, a similar increase in PLN Foxp3^+^ Tregs was not induced in NOD mice treated at 12 weeks of age (Supplemental Figure 6), in which T cells eventually reseeded the islets. One intriguing possibility that may explain these disparate outcomes is that the tolerogenic effect of CoRT can be tuned by local levels of glucose and/or insulin, which impact T cell metabolism and in turn Foxp3^+^ Treg versus Teff differentiation and function (66–68). Interestingly, both glucose and insulin signaling have been shown to affect Foxo1 activity (67, 68).

Reseeding of the pancreas by Teffs permitted discovery of an additional long-term effect of CoRT. The pancreatic CD8^+^ Teff pool was diminished in size and marked by an exhausted phenotype (Figure 10). Importantly, PD-1 blockade induced diabetes in CoRT-protected NOD mice, providing evidence that exhaustion has a functional role in limiting islet Teff pathogenicity (Figure 10). Prior studies have demonstrated that T cell exhaustion limits β cell autoimmunity in NOD mice (36, 37). Furthermore, reduced progression of T1D in patients correlates with an increased frequency of exhausted β cell–specific T cells found in blood (11). Notably, responders to αCD3 therapy in the clinic exhibit an increase in partially exhausted CD8^+^ T cells in blood (69). In addition, preservation of endogenous insulin production in T1D patients receiving alefacept, a CD2-blocking Ab, correlates with an increase in CD8^+^ T cells displaying an exhausted phenotype, marked by PD-1 and Eomes expression (70). Similar to CD4 and CD8, CD2 is a TCR signaling costimulatory molecule. Therefore, these results and our own reinforce the concept that CoRT-like therapies can functionally reprogram pathogenic Teffs and limit T1D progression.

Efforts are currently ongoing to define the mechanism by which CoRT mediates exhaustion within pancreatic CD8^+^ Teffs. Notably, Foxo1 has been linked to CD8^+^ T cell exhaustion (35). Foxo1 regulates expression of PD-1 and Eomes, both of which are upregulated on pancreatic CD8^+^ Teffs after CoRT. One scenario explaining the intrinsic effect of CoRT is that ND αCD8α binding to CD8^+^ T cells results in enhanced Foxo1 activity, which imprints long-lasting epigenetic changes that readily favor exhaustion upon chronic self-antigen stimulation (71). Whether the function of pancreatic CD4^+^ Teffs, which did not exhibit an increase in exhaustion, is intrinsically impacted by ND αCD4 also needs to be further examined. An altered CD4^+^ Teff function may also indirectly contribute to CD8^+^ T cell dysfunction (72, 73). Indeed, single-cell RNA-Seq data of T cells infiltrating the pancreas 60 days after CoRT indicate that CD4^+^ T follicular helper (Tfh) cells are diminished (data not shown). Notably, Tfh cells are a key source of IL-21, which plays a role in regulating CD8^+^ Teff exhaustion (74, 75). Finally, Foxp3^+^ Tregs, which also reseeded the islets, may also in part contribute to reduced Teff pathogenicity.

In summary, our findings provide mechanistic insight into the robust tolerogenic properties of CoRT. Through regulation of TCR signaling, CoRT alters the transcriptional signature of tissue-resident Teffs. The latter is characterized by upregulation of the Foxo1 transcriptional axis, which plays a key role in promoting both Teff tissue egress and the suppression of proinflammatory function. The robust therapeutic efficacy of CoRT is likely due to targeting multiple aspects of T cell biology regulated by Foxo1 (28, 71). Furthermore, a short course of CoRT has persistent intrinsic effects that promote selective exhaustion in pancreatic CD8^+^ Teffs and long-term diabetes prevention. The altered pathogenicity seen in pancreatic CD8^+^ Teffs underscores the need for administering both ND αCD4 and αCD8α to induce and maintain an effective tolerogenic response. Indeed, diabetes remission is induced only with coinjection of ND αCD4 and αCD8α (25). To date, a small number of ND αCD4 Abs have been tested in the clinic for autoimmune diseases such as rheumatoid arthritis, multiple sclerosis, and psoriasis, and to our knowledge no ND αCD8 Ab has been clinically tested (76–83). Based on our findings, we argue that the combination of ND αCD4 and αCD8α is needed to establish robust/optimal induction and maintenance of self-tolerance.

## Methods

### Mice.

NOD/ShiLtJ, NOD.Cg-Tg(TcraBDC2.5)1Doi Tg(TcrbBDC2.5)2Doi/DoiJ (BDC), NOD.Cg-Tg(TcraTcrbNY8.3)1Pesa/DvsJ (8.3), and NOD.Cg-Rag1^tm1Mom^ Il2rg^tm1Wjl^/SzJ (NRG) mice were obtained from the Jackson Laboratory and bred in house. Foxo1^tm1rdp^/J (*Foxo1^fl/fl^*), B6.129P2-S1pr1^tm1hrose^/J (S1PR1-GFP), and Tg(Nr4a1-EGFP/cre)B6-820Khog (Nur77-GFP) were purchased from the Jackson Laboratory, backcrossed 4 times onto the NOD background, and then crossed to NOD.BDC mice to generate the BDC.*Foxo1^fl/fl^* and BDC.S1Pr1-GFP lines. NOD.*Lck^Cre^* mice, a gift from Maureen Su (UCLA, Los Angeles, California, USA), were crossed to NOD.BDC mice to generate the BDC.*Lck^Cre^* line. BDC.*Foxo1^fl/fl^* and BDC.*Lck^Cre^* mice were crossed to generate the BDC.*Foxo1^fl/fl^.Lck^Cre^* line. All mice were bred and housed under specific pathogen–free conditions, and littermate controls were used for experiments. Mice were diagnosed with overt diabetes after 2 consecutive blood glucose readings of 250 mg/dL or higher.

NRG mice were injected with 1.0 × 10^7^ to 2.0 × 10^7^ PBMCs prepared by Ficoll-Paque Plus (GE Healthcare). To monitor reconstitution of NRG.PBL mice, the frequency of HuCD3^+^ cells in peripheral blood was measured via FACS. Cohorts of 3–5 NRG.PBL mice showing 45% or greater peripheral blood CD3^+^ T cells, typically 4 to 6 weeks after PBMC transfer, were used for experimentation.

### Treatments.

YTS177 and YTS105 hybridomas were a gift from Herman Waldmann (University of Oxford, Oxford, UK). Purified YTS177 (αCD4; rat IgG2a) and YTS105 (αCD8α; rat IgG2a) were produced in house from supernatants of cultured hybridomas, as previously described (24). Rat IgG2a control Ab (clone 2A3) was purchased from BioXCell. CH5g5 (αhuCD4; huIgG4) and CH9d2 (αhuCD8α; huIgG4) were purified from Expi293 cell transfectants by protein A affinity chromatography (Hi-Trap Protein A, GE Healthcare), as previously described. Human isotype control polyclonal human IgG was purchased from BioXCell.

Mice were injected i.p. with 700–1,000 μg of YTS177 or YTS105, or 1,400–2,000 μg of 2A3. In some experiments, mice received 2 i.p. injections of YTS177 and YTS105, with or without 500 μg of αPD-1 Ab (clone J43, BioXCell). In other experiments, mice received i.v. injection of sBDC peptide (Anaspec), and PLN and pancreatic T cells were isolated. Humanized NRG.PBL mice were injected i.p. with 500 μg of CH5g5 and 500 μg of CH9d2 or 1.0 mg of polyclonal human IgG (BioXCell). Multiple donors were recruited for NRG.PBL mouse experiments, with each donor generating multiple replicate mice. Replicate mice for a given donor were averaged to generate data points for each treatment group to mitigate variation in NRG.PBL humanization and donor responses.

FTY720 (Sigma-Aldrich) was administered at 1 mg/kg 24 hours before CoRT, and additional injections were given at the time of CoRT and once daily afterwards.

### FACS and T cell enumeration.

Indicated tissues were homogenized into single-cell suspensions, splenic RBCs lysed using Tris-buffered ammonium chloride, and samples filtered for debris. Pancreatic T cells were processed and FACS stained in the presence of 10 mM EDTA to prevent cell aggregation. Reactive Blue (Thermo Fisher Scientific) or Aqua LIVE/DEAD (Invitrogen) discriminator dyes were used to identify viable cells per the manufacturer’s instructions (Life Technologies). Cell surface staining was done on live cells unless otherwise noted. The following FACS Abs were purchased from Invitrogen, BioLegend, Millipore, Cell Signaling Technology (CST), Miltenyi Biotec, or BD Biosciences (BD): αCD45–Brilliant Violet 421 (clone 30-F11, BioLegend); αCD4-PE or -APC (clone RM4-4, BioLegend); αCD3ε-FITC or -PE/Cy7 (clone 145-2C11, Invitrogen); αTCRβ–Brilliant Violet 421 or –Alexa Fluor 594 (clone H57-957, BioLegend); αCD8α–Brilliant Violet 421 (clone 53-6.7, Invitrogen); αCD8β–Brilliant Violet 421 (clone eBioH35-17.2, Invitrogen); αCD127-PE (clone SB/199, Invitrogen); αCCR7-PE/Cy7 (clone 4B12, Invitrogen), αCD69–PerCP-Cy/5.5 (clone H1.2F3, BD); αCD44–PerCP-Cy/5.5 (clone IM7, BD); αCD62L-PE/Cy7 (clone MEL-14, Invitrogen); αCXCR3-APC (clone CXCR3-173, BioLegend); αCXCR4-PE (clone L276F12, BioLegend); αPD-1–PE/Cy7 (clone 29F.1A12, BioLegend); αLAG-3 (clone 4-10-C9, Millipore); αTIM-3–PE (clone 5D12, BD); and αCD103-APC (clone 2E7, Invitrogen). The following anti-human Abs were used for FACS: αCD3-FITC (clone OKT3, BD); αCD4–Brilliant Violet 421 (clone OKT4, BioLegend); αCD8β-PE/Cy7 (clone SIDI8BEE, Invitrogen); αCD69-PerCP-Cy/5.5 (clone FN50, BioLegend); and αCD127-PE/Cy7 (clone A019D5, BioLegend). Surface staining was performed on ice for 30 minutes, after which cells were fixed. Fixation of cells for intracellular transcription factors was done using eBio Fix/Perm reagents per the manufacturer’s instructions. Fixation of GFP reporter mice or intracellular staining for cytokines was carried out using BD Fix/Perm reagents. Fixation of cells for phospho-FACS was performed after the indicated stimulation times using 2% paraformaldehyde for 15 minutes at room temperature followed by permeabilization in 100% methanol for 30 minutes at –20°C. For ImageStream flow cytometry, cells were isolated from the PLN and immediately fixed in eBio Fix/Perm reagents per the manufacturer’s instructions. Cells were then stained for surface and intracellular proteins, and with the DNA-staining dye Hoechst (Thermo Fisher Scientific). The following Abs were used for intracellular staining: αT-bet–Alexa Fluor 488 (clone 4B10, BioLegend); αIRF4-PE (clone IRF4.3E4, BioLegend); αFoxo1 (clone C29H4, CST); αp-Foxo1 (clone 9464, CST); αp-ZAP70 (clone 65E4, CST), αp-AKT Ser473 (clone D9E, CST); αp-AKT Thr 308 (clone D25E6, CST); αIFN-γ–FITC (clone XMG1.2, BD); αIL-2–APC (clone JES6-5H4, Invitrogen); αIL-17–PE (clone eBio17B7, Invitrogen); αTNF-α–PE/Cy7 (clone MP6-XT22, BD); αLCK (catalog 2752, CST); αKi67-APC (clone B56, BD); αEomes-PE (clone Dan11mag, Invitrogen); αTOX-PE (clone REA473, Miltenyi Biotec); and αFoxP3-FITC (clone FJK-16s, Invitrogen). Non–fluorochrome-conjugated Abs were stained with goat anti-rabbit–Alexa Fluor 647 (Invitrogen).

T cells were enumerated using AccuCheck counting beads according to the manufacturer’s instructions (Life Technologies). FACS data were acquired using a BD LSRII or a BD Fortessa, and data were analyzed using FlowJo software (TreeStar). ImageStream flow cytometry data were acquired using the ImageStream X Mark II, and data were analyzed using Ideas image analysis software.

### Cell enrichment/cell sorting.

Murine CD4^+^ or CD8^+^ cells were flow sorted using a BD FACSAria III. Murine and human CD4^+^ and CD8^+^ T cell enrichment was performed on an AutoMACS separator using a CD4^+^ or CD8^+^ T cell isolation kit according to the manufacturer’s specifications (Miltenyi Biotec).

### In vitro assays.

Isolated splenocytes or PLN-derived cells were cultured in a 96-well round bottom plate (Corning), stimulated with 0–10 μg/mL sBDC (Anaspec) or IGRP_206–214_ (Anaspec), pelleted, and then incubated at 37°C for 30–60 minutes for subsequent phospho- or ImageStream flow cytometry. In some experiments, cells were cultured in a round-bottom 96-well plate and stimulated 4 hours with PMA (1:2000; Sigma-Aldrich) and ionomyocin (1:1000; Sigma-Aldrich) or 1 μg/mL sBDC (Anaspec) in the presence of brefelden A (1:1,000; BioLegend), monensin (1:1,000; BioLegend), and eBioscience protein transport inhibitor cocktail (1:500; Invitrogen).

### Microarray analysis, quantitative real-time PCR, and primers.

PLN from 3 BDC mice were pooled and CD4^+^ T cells were sorted by FACS. RNA for microarray analysis was prepared using RNeasy PLUS reagents (QIAGEN). Mouse expression 2.0 ST arrays from Affymetix were used for the microarray assay and analysis by the Genomics Core Facilities at the University of North Carolina at Chapel Hill. The microarray data have been deposited in NCBI’s Gene Expression Omnibus database under accession number GSE179650.

RNA for qRT-PCR was prepared from isolated cells using an RNeasy PLUS kit (QIAGEN), and cDNA synthesized using Maxima H Minus First-Strand cDNA synthesis kit (Thermo Fisher Scientific) according to the manufacturer’s recommendations. cDNA was amplified via a Maxima SYBR Green master mix (Thermo Fisher Scientific). Ct values were calculated by the ΔΔCt method and normalized to *HPRT* values. The following murine PCR primers were used: *HPRT* Fwd, 5′-GCTATAAATTCTTTGCTGACCTGCTG-3′; *HPRT* Rev, 5′-AATTACTTTTATGTCCCCTGTTGACTGG-3′; *Foxo1* Fwd, 5′-TGTCAGGCTAAGAGTTAGTGAGCA-3′; *Foxo1* Rev, 5′-GGGTGAAGGGCATCTTTG-3′; *Ccr7* Fwd, 5′-TGATTTCTACAGCCCCCAGA-3′; *Ccr7* Rev, 5′-GCACACCTGGAAAATGACAA-3′; *Cd127* (IL-7ra) Fwd, 5′-GCGGACGATCACTCCTTCTG-3′; *Cd127* (IL-7ra) Rev, 5′-AGCCCCACATATTTGAAATTCCA-3′; *Klf2* Fwd, 5′-ACCAACTGCGGCAAGACCTA-3′; *Klf2* Rev, 5′-CATCCTTCCCAGTTGCAATGA-3′; *Sell* (CD62L) Fwd, 5′-CTAATTTCCCCTCGCTCATTCAT-3’; *Sell* (CD62L) Rev, 5′-GCATTAGCTTCTGTGCTGAATTGA-3′; *S1pr1* Fwd, 5′-GTGTAGACCCAGAGTCCTGCG-3′; *S1pr1* Rev, 5′-AGCTTTTCCTTGGCTGGAGAG-3′; *Cd69* Fwd, 5′-TGGTCCTCATCACGTCCTTAATAA-3′; *Cd69* Rev, 5′-TCCAACTTCTCGTACAAGCCTG-3′; *Irf4* Fwd, 5′-CAAAGCACAGAGTCACCTGG-3′; *Irf4* Rev, 5′-TGCAAGCTCTTTGACACACA-3′; *Nr4a1* (Nur-77) Fwd, 5′-CGGACAGACAGCCTAAAAGG-3′; *Nr4a1* (Nur-77) Rev, 5′-TAACGTCCAGGGAACCAGAG-3′; *Tbx21* (T-bet) Fwd, 5′-AGCAAGGACGGCGAATGTT-3′; *Tbx21* (T-bet) Rev, 5′-GGGTGGACATATAAGCGGTTC-3′; *Cd25* (IL-2ra) Fwd, 5′-GAGTGAGACTTCCTGCCCCATA-3′; *Cd25* (IL-2ra) Rev, 5′-TCTCCGTCATTGCAGTTGTTT-3′; *Cd3ε* Fwd, 5′-GCTACACACCAGCCTCAAATA-3′; *Cd3ε* Rev, 5′-CAAGCCCAGAGTGATACAGATG-3′. The following human PCR primers were used: *GAPDH* Fwd, 5′-AATCCCATCACCATCTTCCA-3′; *GAPDH* Rev, 5′-TGGACTCCACGACGTACTCA-3′; *Foxo1* Fwd, 5′-GCCATGTAAGTCCCATCAGGA-3′; *Foxo1* Rev, 5′-ATCGGAACAAGAACGTGGAATC-3′; *CCR7* Fwd, 5′-GCCAACTTCAACATCACCA-3′; *CCR7* Rev, 5′-AAGGCGTACAAGAAAGGGTT-3′; *CD127* Fwd, 5′-CCCTCGTGGAGGTAAAGTGC-3′; *CD127* Rev, 5′-CCTTCCCGATAGACGACATCT-3′; *KLF2* Fwd, 5′-CACCGGGTCTACACTAGAGG-3′; *KLF2* Rev, 5′-AAATGCCGCAGACAGTACAA-3′; *Sell* (CD62L) Fwd, 5′-CTGGCACATCATGGAACCGAC-3′; *Sell* (CD62L) Rev, 5′-GTGTAATTGTCTCGGCAGA-3′; *S1PR1* Fwd, 5′-TTCTGCGGGAAGGGAGTATGT-3′; *S1PR1* Rev, 5′-AAGAGGCGGAAGTTATTGCT-3′; *CD3ε* Fwd, 5′-GGCAAAGGGGACAAAACAAG 3′; *CD3ε* Rev, 5′-CTTTCCGGATGGGCTCATAG-3′.

### Histology and microscopy.

Paraffin-embedded pancreatic sections were stained with hematoxylin and eosin. Bright-field images were obtained with an Olympus BX61 microscope with a 40×/0.75 UPlanFLN objective lens and a RETIGA 4000R camera. Images were acquired by Improvision’s Velocity software s at 3.4 megapixel resolution (1848 × 1848 pixels). Images were processed using FIJI software and flat-field correction was done via macros created by Robert Bagnell and Pablo Ariel (Microscopy Core Facility of the University of North Carolina at Chapel Hill) (84). Insulitis was scored in a blinded manner; a minimum of 50 islets for each individual pancreas were examined.

### Statistics.

Data on scatter and line plots appear with the mean identified by a horizontal line. Error bars represent SD. For experiments containing 2 groups, 2-tailed Student’s *t* test was used to determine significance. For experiments containing more than 2 groups, 2-way analysis of variance (ANOVA) with Bonferroni’s multiple comparison correction was used to determine significance. ImageStream data are represented by SI calculated by Ideas software measuring colocalization by similarity of nucleus-stained images and transcription factor–stained images. A *P* value of less than 0.05 was considered significant: *P < 0.05, **P < 0.01, ***P < 0.001.

### Study approval.

All animal procedures were approved by the University of North Carolina at Chapel Hill (UNC-CH) IACUC. Human PBMCs were collected from healthy donors in accordance with UNC-CH IRB–approved guidelines.

## Author contributions

MC and RT designed research. MC, BW, CJK, QK, AM, KS, and RZ performed research. MC, RT, BW, AM, and JJM analyzed data. MC and RT wrote the manuscript.

## Supplementary Material

Supplemental data

## Version 1 07/27/2021

In-Press Preview

## Version 2 09/08/2021

Electronic publication
