# Supplementary material for: Coreceptor therapy has distinct short- and long-term tolerogenic effects intrinsic to autoreactive effector T cells
Source: JCI Insight. 2021 Sep 8;6(17):e149130. doi: 10.1172/jci.insight.149130 (PMC8492310; doi:10.1172/jci.insight.149130)
Supplement: Supplemental data [file jciinsight-6-149130-s031.pdf]

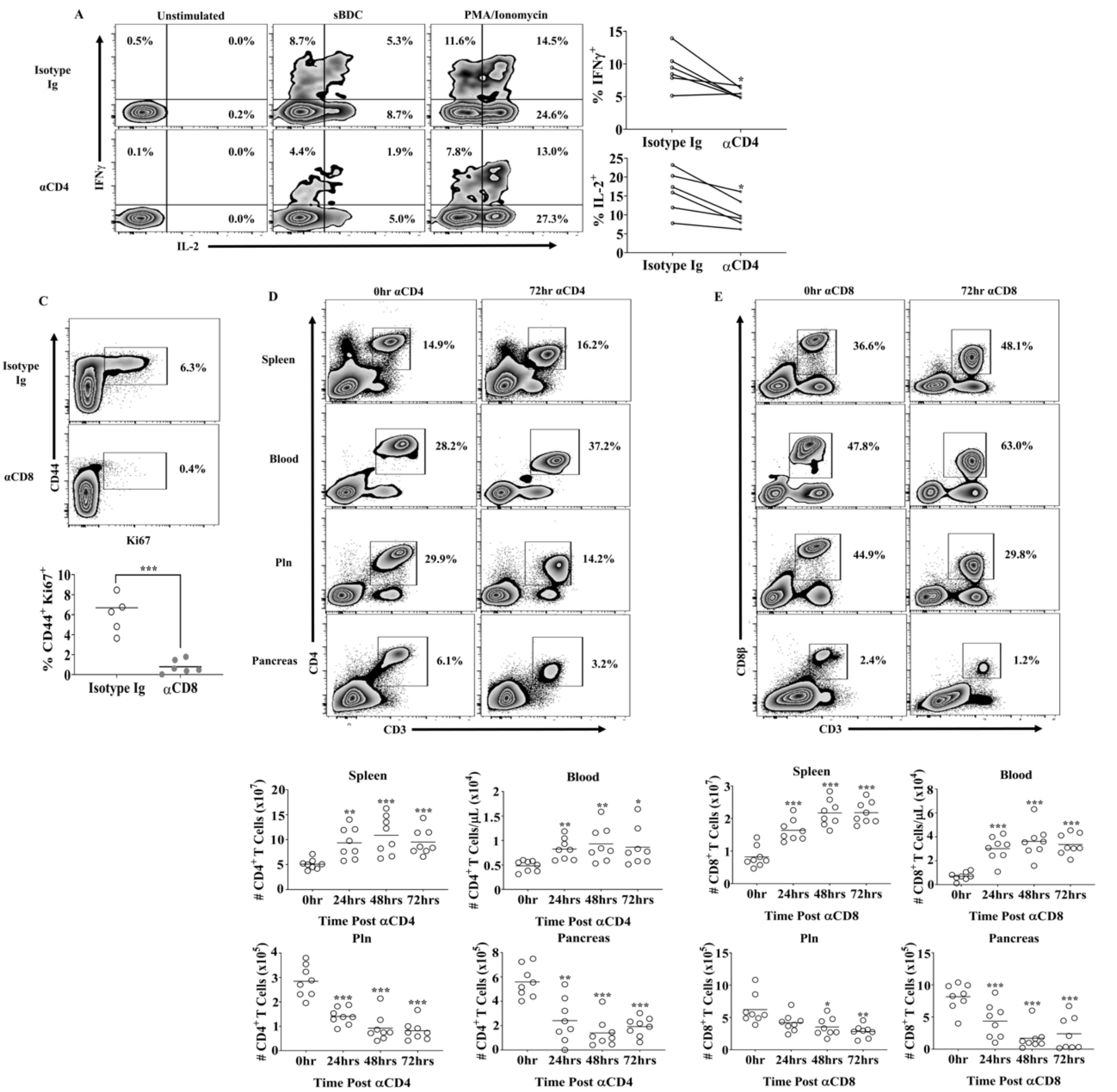

Supplemental FIGURE 1. ND αCD4/CD8 Ab treatment induces T1D remission and T cell egress from inflamed tissues. A) BDC mice (N=6) were treated with ND αCD4 or control Ab, 24 hrs later splenocytes stimulated with 1μg sBDC peptide *in vitro* or PMA/Ionomycin for 4 hrs, and CD4+ T cells assessed for intracellular staining by FACS. Plots provide the percent of IL-2+ and IFNγ+ T cells stimulated with peptide and treated with Ab. **B)** BDC and **C)** 8.3 mice (N=6) received ND αCD4 or αCD8α, respectively, and PLN T cells were analyzed 48 hrs later by FACS. **D)** BDC and **E)** 8.3 mice (N=8) received ND αCD4 or αCD8α, respectively, and T cells enumerated by FACS in the spleen, PLN, and pancreas at various times post-treatment. P value determined by Students t-test (**A-C**) or 2-way ANOVA (**D, E**). Error bars depict SD. *P<0.05, **P<0.01, ***P<0.001. Data are pooled from 2 experiments.

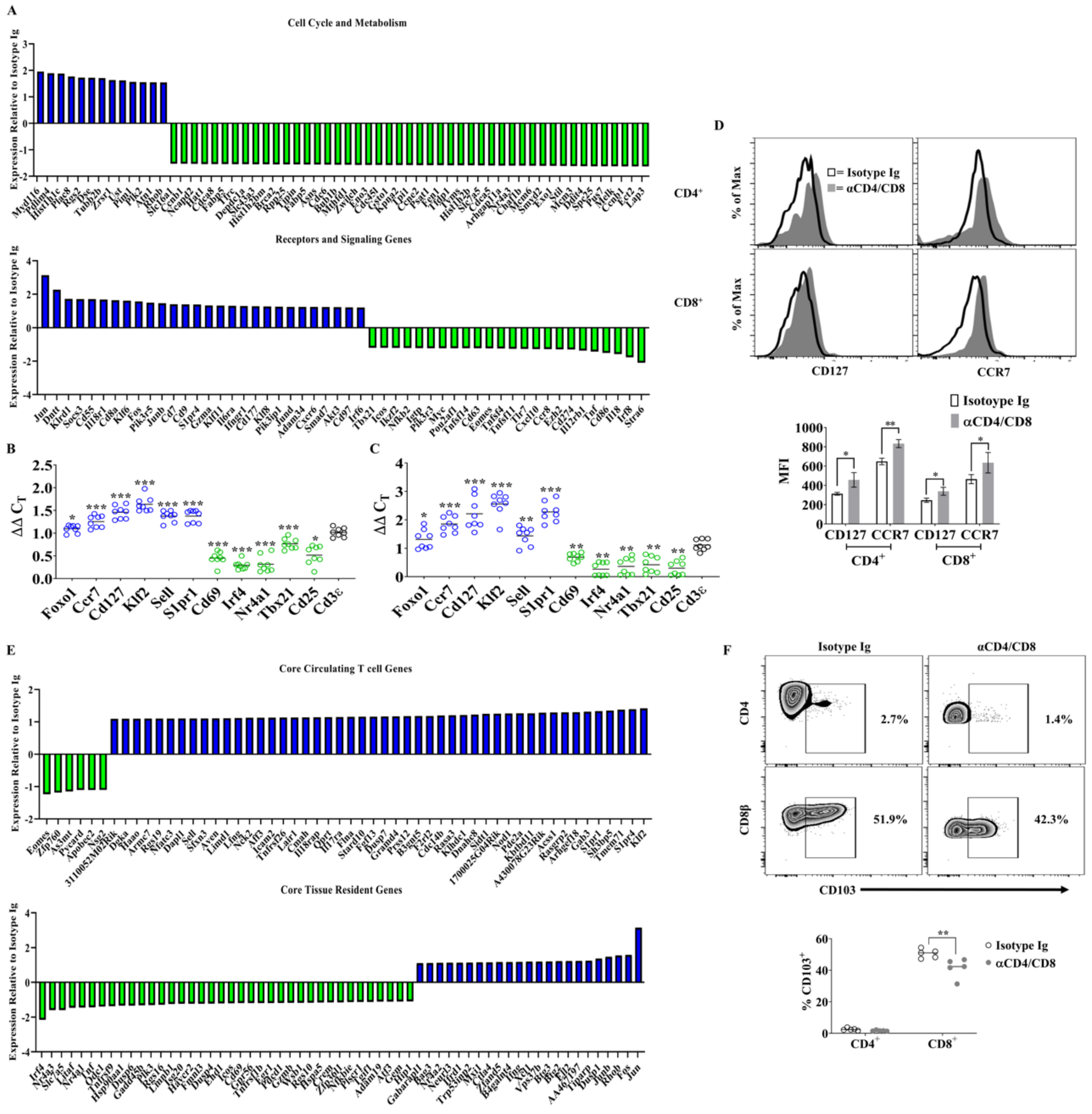

Supplemental FIGURE 2. Foxo1 regulated gene expression is increased in T cells after ND αCD4/CD8 Ab treatment. A, E) Microarray analysis of FACS-sorted CD4^{+} T cells from the PLN of BDC mice 48 hrs after ND αCD4 or control Ab treatment. Values relative to the Ab control cohort. **B, C)** mRNA expression measured by qRT-PCR in (**B**) CD4^{+} and (**C**) CD8^{+} T cells isolated from the pancreas of NOD mice (N=8) 24 hrs after receiving ND αCD4/CD8α or control Ab. ΔΔCT calculated relative to Ab control-treated CD4^{+} or CD8^{+} T cells (Foxo1 related genes in blue; T cell activation genes in green). **D, E, F)** 12wk-old NOD female mice (N=5) were treated with ND αCD4/CD8α or isotype control Ab, and pancreatic T cells analyzed 48 hrs later by FACS. P value determined by Students t-test (**B-D, F**). Error bars depict SD. *P<0.05, **P<0.01, ***P<0.001. Data are pooled from 2 experiments.

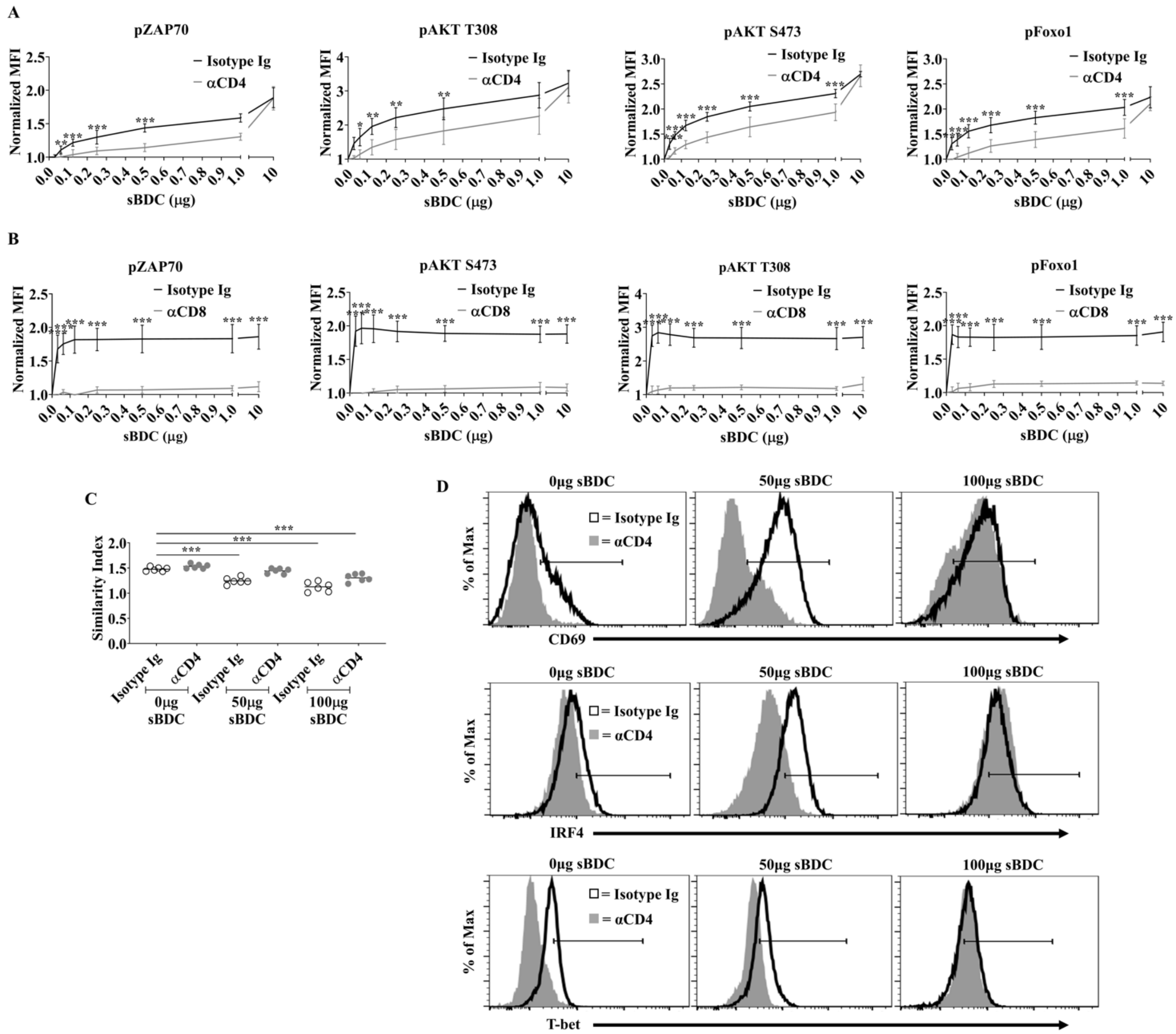

Supplemental FIGURE 3. TCR signaling-dependent pancreatic T cell tissue residency is lost upon CoRT induced Foxo1-S1pr1. A) BDC and **B)** 8.3 mice were treated with ND αCD4 and αCD8α, respectively, or control Ab, 24 hrs later splenocytes isolated and stimulated *in vitro* for 30 min (pZAP70) or 1 hr (pAKT/Foxo1) with cognate peptide (N=6), and TCR signaling assessed by FACS. **C, D)** BDC mice were treated with ND αCD4 or control Ab (N=6), immunized with sBDC **(C)** 18 hrs later, and after 1 hr PLN T cells examined by image stream flow cytometry, and **(D)** 6 hrs later, and after 18 hrs pancreatic $CD4^+$ T cells examined by FACS. Error bars depict SD. *P<0.05, **P<0.01, and ***P<0.001 determined by Students t-test (**A, B**) or 2-way ANOVA (**C, D**). Data are pooled from 2 to 3 experiments.

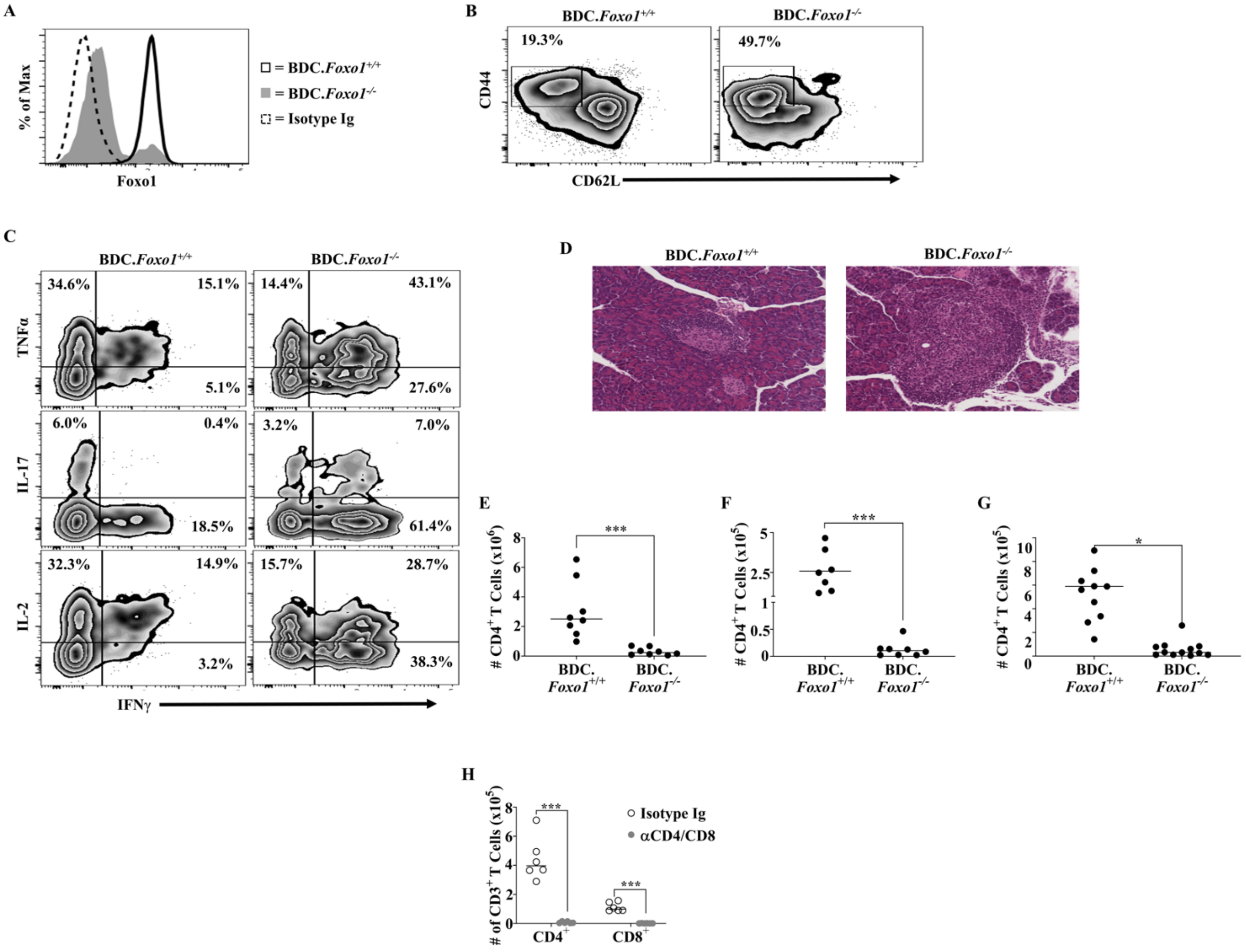

Supplemental FIGURE 4. CoRT-induced T cell egress is Foxo1-dependent. A and B) Gated splenic $CD4^+$ T cells from BDC.*Foxo1*$^{+/+}$ and BDC.*Foxo1*$^{-/-}$ mice were assessed for (**A**) intracellular Foxo1 expression, and (**B**) activation status. **C)** Gated splenic $CD4^+$ T cells from BDC.*Foxo1*$^{+/+}$ and BDC.*Foxo1*$^{-/-}$ mice were assessed for intracellular cytokine expression 4 hrs after PMA/ionomycin treatment by FACS. **D)** Representative pancreas sections stained with hematoxylin and eosin from BDC.*Foxo1*$^{+/+}$ and BDC.*Foxo1*$^{-/-}$ mice. **E, F, G)** Splenic (N=8) (**E**), PLN (N=8) (**F**), and pancreatic (N=10) (G) $CD4^+$ T cells in BDC.*Foxo1*$^{+/+}$ and BDC.*Foxo1*$^{-/-}$ mice were enumerated by FACS. **H)** NOD.*Lck*^{Cre} mice (N=6) were treated with ND αCD4/CD8α or control Ab, and 72 hrs later pancreatic $CD4^+$ and $CD8^+$ T cells enumerated by FACS. P value determined by Students t-test (**E-H**). Error bars depict SD. *P<0.05, **P<0.01, ***P<0.001. Data are pooled from a minimum of 3 experiments.

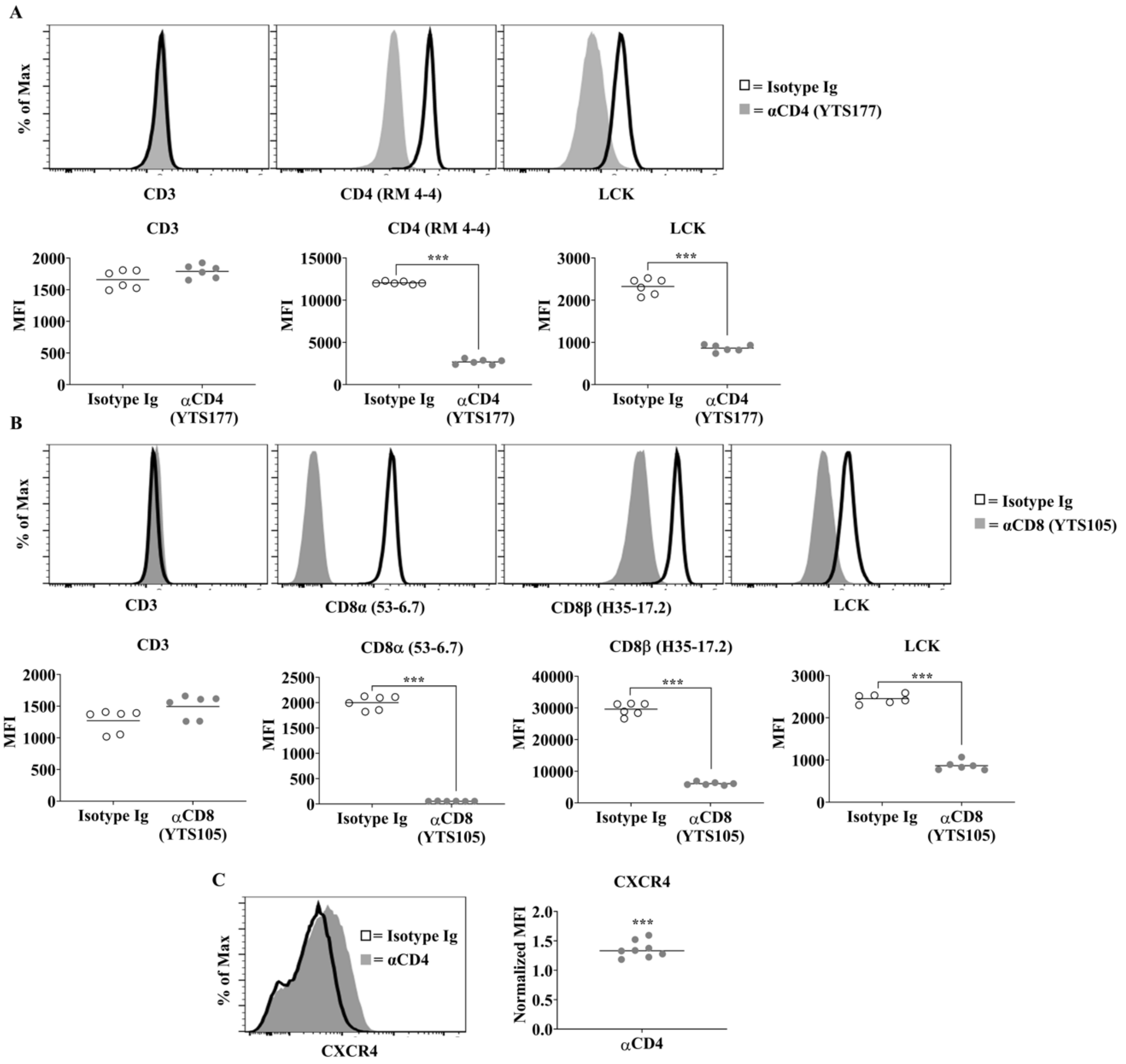

Supplemental FIGURE 5. CoRT decreases CD4 and CD8 surface expression, and intracellular Lck. A) BDC and (**B**) 8.3 mice (N=6) were treated with ND αCD4 and ND αCD8α, respectively, or control Ab, and 48 hrs later splenocytes isolated. Gated (**A**) $CD4^+$ and (B) $CD8^+$ T cells were examined by FACS for surface expression of CD3, CD4 (RM 4-4), CD8α (53-6.7), and CD8β (H35-17.2), or intracellular Lck. **C)** BDC mice (N=8) were treated with ND αCD4 (YTS177) or isotype control Ab, 48 hrs later splenocytes were isolated and analyzed by FACS. P value determined by Students t-test (**A-C**). Error bars depict SD. *P<0.05, **P<0.01, ***P<0.001. Data are pooled from 2 experiments.

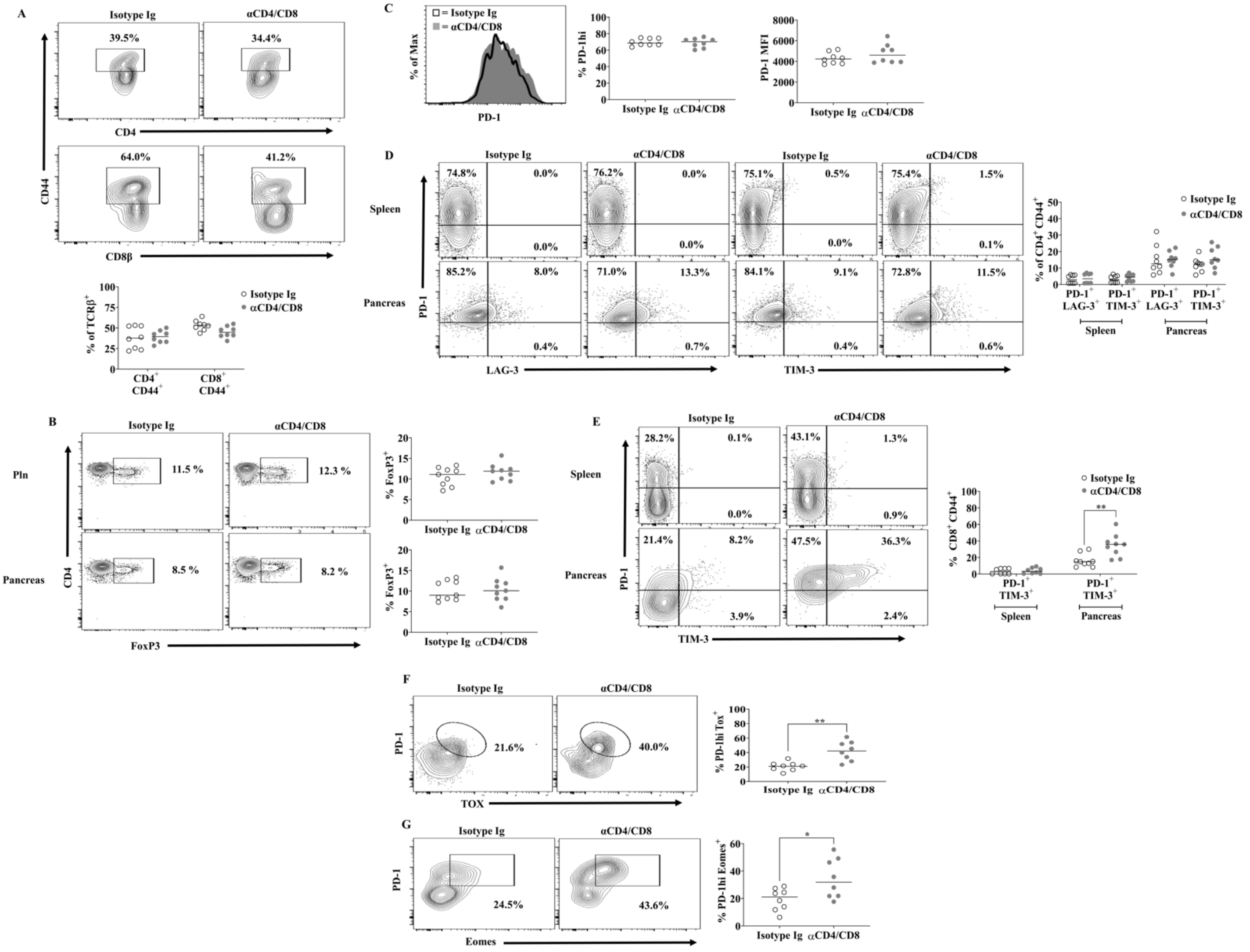

Supplemental FIGURE 6. ND αCD4/CD8 Ab treatment promotes selective CD8^{+} Teff exhaustion. A-D) 12 wk-old NOD female mice received 2 injections one day apart with ND αCD4/CD8α or isotype control Ab, and 60 d later T cells examined via FACS. **A)** Percent of pancreatic CD44^{+} CD4^{+} and CD8^{+} T cells in ND αCD4/CD8α (N=8) or control Ab (N=8) groups. **B**) Frequency of pancreatic and PLN CD4^{+} T cells expressing FoxP3 in ND αCD4/CD8α (N=8) or control Ab (N=8) groups. **C-D)** Frequency of pancreatic and splenic CD44^{+} CD4^{+} Teff co-expressing PD-1 and LAG-3 or TIM-3 in ND αCD4/CD8α (N=8) or control Ab (N=8) groups. **E-G**) Pancreatic and splenic CD44^{+} CD8^{+} Teff were examined by FACS for PD-1 and co-expression of TIM-3, TOX and Eomes 60 d post treatment with ND αCD4/CD8α (N=8) or control Ab (N=8). P value determined by Students t-test (**A-G**). Error bars depict SD. *P<0.05, **P<0.01, ***P<0.001. Data are pooled from 2 experiments.
